# Optimizing the Personalized Care for the Management of Rectal Cancer: A Consensus Statement

**DOI:** 10.5152/tjg.2022.211103

**Published:** 2022-08-01

**Authors:** Erman Aytaç, Leyla Özer, Bilgi Baca, Emre Balık, Yersu Kapran, Orhun Cığ Taşkın, Başak Oyan Uluç, Mehmet Ufuk Abacıoğlu, Murat Gönenç, Yasemin Bölükbaşı, Barbaros E. Çil, Bülent Baran, Cem Aygün, Mehmet Erdem Yıldız, Kemal Ünal, Burçak Erkol, Tunç Yaltı, Uğur Özbek, Tan Attila, Nurdan Tözün, Bengi Gürses, Sibel Erdamar, Özlem Er, Nuran Beşe, Orhan Bilge, Güralp Onur Ceyhan, Nil Molinas Mandel, Uğur Selek, Cengiz Yakıcıer, Hülya Kayserili Karabey, Murat Saruç, Volkan Özben, Eren Esen, Emre Özoran, Erkan Vardareli, Levent Güner, İsmail Hamzaoğlu, Dursun Buğra, Tayfun Karahasanoğlu, The İstanbul Group

**Affiliations:** 1 Acıbadem Mehmet Ali Aydınlar University Faculty of Medicine, İstanbul, Turkey; 2 Koç University Faculty of Medicine, İstanbul, Turkey

**Keywords:** Consensus, evidence-based data, rectal cancer, treatment

## Abstract

Colorectal cancer is the third most common cancer in Turkey. The current guidelines do not provide sufficient information to cover all aspects of the management of rectal cancer. Although treatment has been standardized in terms of the basic principles of neoadjuvant, surgical, and adjuvant therapy, uncertainties in the management of rectal cancer may lead to significant differences in clinical practice. In order to clarify these uncertainties, a consensus program was constructed with the participation of the physicians from the Acıbadem Mehmet Ali Aydınlar and Koç Universities. This program included the physicians from the departments of general surgery, gastroenterology, pathology, radiology, nuclear medicine, medical oncology, radiation oncology, and medical genetics. The gray zones in the management of rectal cancer were determined by reviewing the evidence-based data and current guidelines before the meeting. Topics to be discussed consisted of diagnosis, staging, surgical treatment for the primary disease, use of neoadjuvant and adjuvant treatment, management of recurrent disease, screening, follow-up, and genetic counseling. All those topics were discussed under supervision of a presenter and a chair with active participation of related physicians. The consensus text was structured by centralizing the decisions based on the existing data.

## Introduction

Colorectal cancer (CRC) is the third most common cancer in Turkey.^1^ Management of rectal cancer consists of a combination of neoadjuvant therapy, surgery, and adjuvant treatment. However, the current guidelines do not cover all aspects of management of rectal cancer, especially for the diagnosis of rectal cancer, defining organ preservation strategies, selection of proper neoadjuvant modalities, surgical treatment for the primary disease, the role of adjuvant therapy, management of recurrent disease, screening, follow-up, and genetic counseling. Thus, a lack of standardization may lead to significant differences in clinical practice. This consensus program aimed to establish feasible, logical, measurable, and collective solutions to challenges that our participant physicians from 2 leading academic institutions face during the management of rectal cancer. Secondly, we aimed to emphasize that multi-disciplinary tumor boards (MDTB) should be mandated before any management decision is made regarding the treatment of the rectal cancer.

## MATERIALS AND Methods

One hundred twenty-seven physicians from the departments of gastroenterology, general surgery, genetics, medical oncology, nuclear medicine, radiation oncology, radiology, and pathology of Acıbadem Mehmet Ali Aydınlar and Koç Universities organized a consensus program to focus on the management of rectal cancer. This consensus program included management of rectal adenocarcinoma solely. Other histologic types of rectal cancers were excluded. A board committee was assigned to define the gray zones in the management of rectal cancer by reviewing the evidence-based data and current guidelines. This committee consisted of at least 1 representative from each department. Topics regarding diagnosis, staging, surgical treatment of primary disease, use of neoadjuvant and adjuvant therapy, management of recurrent disease, screening, follow-up, and genetic counseling were determined by the committee for discussion. These topics were discussed, voted, and ratified statement by statement under the supervision of a presenter and a chair along with the participation of a large group of physicians. Unanimously agreed statements were included in the consensus paper. Eighth version American Joint Committee on Cancer Union for International Cancer Control/Tumor-Node-Metastasis (AJCC-UICC/TNM) classification was used for staging.

### Presentation, Diagnosis, and Local Management for Primary Disease

The reported incidence of CRC in Turkey is 13-22 cases/100 000 population per year and is predicted to increase. Approximately, 30% of colorectal tumors originate from the rectum. Clinical investigations have shown that epidemiology, etiology, and risk factors of rectal cancer differ from that of colon cancer.^2-4^

### Diagnosis of Rectal Carcinoma

Rectal cancer is categorized as low (up to 5 cm from the anal verge), middle (between 5 and 10 cm from the anal verge), or high (between 10 up to 15 cm from the anal verge) according to its location. Diagnosis of rectal cancer is established by colonoscopy and biopsy.^5^ The lower rectum, anal canal, and prostate gland can be examined by digital rectal examination (DRE). However, DRE has a low sensitivity as a screening evaluation since it may be associated with high false-negative results.^6^ High-resolution optical methods such as narrow-band imaging, laser confocal endoscopy, or chromoendoscopic methods using dye solutions can be used to identify high-risk, flat premalignant lesions or early-stage carcinomas. Flat or depressed lesions carry a higher risk of in situ or invasive carcinoma. A complete colonoscopy should be performed to rule out synchronous tumors, as well as polyps at the rest of the colon, after finding a suspicious lesion.

### Use of Rigid Rectoscopy

The current gold standard for the detection of colorectal cancer is a flexible colonoscopy. A rigid rectoscopy may have the advantage of taking deeper and larger biopsies.^7^

### Use of an Endoscopic Ruler

The experience of an endoscopist is the most important determinant for the accurate determination of lesion size.^8^ The use of an endoscopic ruler may enable endoscopists to measure and describe rectal cancer more accurately.

### Role of Carcinoembryonic Antigen in a Clinical Setting

Carcinoembryonic antigen (CEA) allows effective disease monitoring of CRC during adjuvant treatment and postoperative follow-up. The European Group on Tumor Markers, European Society of Medical Oncology, and American Society of Clinical Oncology guidelines do not recommend CEA as a screening test. Elevated CEA concentrations in patients with stage II and III CRC were found to be associated with aggressive behavior of cancer. From a prognostic point of view, it is reasonable to monitor CEA levels after diagnosis of rectal cancer for detection of recurrences.^9-11^

## Imaging and Preoperative Staging

### Standard Imaging Modality

*A magnetic resonance imaging (MRI) of the pelvis and a thoracoabdominal computed tomography (CT) are the standard imaging modality for staging rectal cancer*. High-resolution pelvic MRI plays a critical role in surgical decision-making since it provides detailed images of mesorectal fascia and its contents. Magnetic resonance imaging has a high specificity (92%) for negative clear resection margin estimation.^12^ It is superior from other modalities in detecting extramural vascular invasion (EMVI), determining T sub-stages, and determining the distance of tumor to mesorectal fascia. Thus, preoperative complete resection margin can be evaluated accurately, and patients can be risk-stratified via MRI.^13^ Magnetic resonance imaging has higher sensitivity and accuracy than endoscopic ultrasonography (EUS) in nodal staging.^14^ Endoscopic ultrasonography is more specific than MRI in the evaluation of muscularis propria invasion; therefore, it should be performed for staging of T1-T2 tumors, prior to planning of local excision.^15,16^ In obstructing cancers, the endoscopic ultrasound scope may not be able to traverse the malignant stricture and therefore may not accurately evaluate the depth of tumor invasion. Therefore, the accuracy of EUS for the staging of T4 tumors ranges between 44% and 50%.^17^ Rather than choosing one against the other, MRI and EUS can be used together as needed for defining the stage of cancer more accurately.^14,17^ Studies on the outcome of dynamic contrast-enhanced (DCE)-MRI after primary and neoadjuvant therapy are inconclusive.^18^

### Magnetic Resonance Imaging Criteria for Pathologic Perirectal Lymph Nodes

Sensitivity of MRI for nodal staging in rectal cancer was found to be only 66%-77% and specificity was 71%-76% in meta-analyses.^16,19,20^ Although the perirectal lymph node involvement is an important factor in the likelihood of metastatic disease, its overall positive predictive value is low.^21^ Magnetic resonance imaging may not identify nodal micro-metastases when the perirectal lymph nodes are smaller than 5 mm.^22^ Approximately, 25% of the lymph nodes were shown to be over-staged.^16^ If any perirectal lymph node is 9 mm or wider on the short axis, it should be reported as suspicious.^15^

*Irregular contour*,* round shape*,* and heterogeneous signal content are the morphological MRI criteria for metastatic perirectal lymph nodes regardless of their size*.^15,22,23^

#### Basic Parameters of an Magnetic Resonance Imaging Report for Local Staging

The report of a high-resolution rectal MRI should be comprehensive during initial staging and after neoadjuvant treatment.^24-27^ Distance between the lowest tumor margin and the anal verge should be included in the report. The size and the circumferential location of the tumor within the wall ought to be described in a clockwise manner.^28^ Describing the tumor location in relation to the anterior peritoneal reflection is important.^28^ Description of T-stage, especially 3 sub-stages to determine the depth of tumor invasion, the nearest distance to mesorectal fascia, and relations with the anal sphincter and levator ani muscles are important prognostic factors.^24^ Rectal MRI reports should include the location and morphology of suspicious nodes, as well as EMVI status.^19,29,30^ The report should be finalized with a cTNM.

## Specific Considerations at the Time of Diagnosis and Staging

### Definition of the Upper Rectum

In order to determine the treatment strategy, differentiation between the distal sigmoid colon and the upper rectal tumor is important.^31^ There is considerable sex and racial variation in the length of rectal and anal canal. *Radiologically*,* the definition of the upper border of rectum varies between S1 and S3 vertebral levels.^32^ The rectosigmoid junction is surgically determined by loss of taenia coli*,* the onset of peritoneal reflection*, *and sacral promontory.^33,34^ Endoscopic and radiologic (MRI) definition of the upper border of rectum varies between 12 and 15 cm from the anal verge*.*^35-39^* However, those definitions do not correlate in a considerable amount of patients. In a study of 128 patients with tumor level determination of sigmoid and rectal cancers, the concordance between endoscopic and radiologic measurement was found to be approximately 80%, and the overall accuracy was 87.5% for endoscopy and 90.5% for imaging.^40^ In case of any discordance in determining the anatomic borders of the rectum, a joint decision should be made to determine management strategy.^41^

### Management of Malignant Polyps

Any polypoid lesion (pedunculated, sessile, or flat) noted during colonoscopy should be completely resected. Pedunculated lesions are removed by snare polypectomy technique. Local recurrence and lymph node metastasis of completely resected pedunculated polyps confined to the superficial submucosa without any unfavorable histopathologic findings are negligible.^42,43^ Therefore, surgery can be omitted in these cases. *For pedunculated polyps with unfavorable histological features (<1 mm cancer-free margin*,* poor histological differentiation*,* vascular or lymphatic invasion)*,* invading the submucosa of the bowel wall below the stalk of a polyp*,* or extending through the submucosa into the deeper wall surgery is recommended*.^44,45^ Endoscopic removal of laterally spreading flat lesions may require more advanced resection techniques.

## Principles of Local Excision for Rectal Cancer Treatment

While endoscopic mucosal resection (EMR) and endoscopic submucosal dissection (ESD) are suggested as effective and safe alternatives to surgery for patients with superficial and early neoplastic lesions of the rectum in selected cases, those procedures may result with positive margin, because during the ESD procedure, the plane for the dissection is mostly between the mucosa and submucosa.^45-48^ Difficulty to elevate the lesion with submucosal injection may be an indicator of submucosal tumor invasion and precludes endoscopic resection. *It is recommended to obtain the lesion in a single piece for an adequate histopathologic evaluation.* Transanal endoscopic surgery (TES) [transanal endoscopic microsurgery (TEM) and a transanal minimally invasive surgery (TAMIS)] can be used for local excision of rectal neoplasms. *In patients with polyps having malignant features according to the endoscopic classifications*,* a proper clinical staging should be performed before procedure.* There is no clear evidence for the full thickness re-excision of the ESD/EMR scar in remnant pT1 lesions.

A diagnostic colonoscopy should not be converted to an advanced endoscopic intervention for treating an early rectal cancer if informed consent is not taken prior to the procedure. A follow-up colonoscopy is recommended in 2-6 months after complete endoscopic removal of a rectal neoplasm.^40,49^ *If the histopathologic evaluation shows malignant features with undetermined resection margin invasion*, *surgery should be considered*.^50^ *Since a lesion with submucosal invasion has a risk of lymph node metastasis ranging from 6% to 12%*,* surgical resection should be considered for endoscopically resected lesions with submucosal invasion*.^51-53^

Accurate histopathologic evaluation of a locally resected specimen is crucial to determine lymph node metastasis risk. *The* *risk of lymph node metastasis of rectal cancer is summarized in* *Table 1*.*^54,55^* As an alternative to radical surgery, a transanal local excision is a favorable option for cT1(sm1)N0M0 rectal cancer without high-grade differentiation or lymphovascular invasion (LVI).^56^ The specimen should be handled cautiously for accurate histopathologic evaluation (depth of invasion, surgical margins, LVI, and differentiation) after transanal local excision. *Radical surgery should be performed for a locally excised lesion with a final pathology reporting pT1sm2 disease*.^35^

### Evaluation of Proximal Colon and Staging in the Setting of Obstructive Rectal Tumors

About 3.5% of the CRCs are synchronous.^57^ *Proximal colon should be assessed with full colonoscopy and abdominopelvic CT with oral and intravenous (IV) contrast at the time of diagnosis.* A completely obstructing rectal tumor may not allow a full colonoscopic evaluation to detect possible synchronous tumors.^58^ In such conditions, post-surgical colonoscopic evaluation within 3-6 months is reasonable.^17,35,59^ There are alternative strategies for synchronous tumor detection for patients requiring emergent surgery.^58^ Preoperatively, abdominal CT with contrast can be performed quickly for diagnosis, and chest CT may be added for staging.^60,61^ In case of incomplete obstruction, CT colonography with rectal air or water is an option.^62-64^ *In patients with proven metastatic and obstructive disease*,* fluorodeoxyglucose (FDG) positron emission tomography/computed tomography (PET/CT) (FDG PET/CT) can be considered to detect other possible sites of metastases. Intraoperative colonoscopy is useful to determine the extent of surgical resection.^58^*

### Imaging Choice of Peritoneal Metastases in Rectal Cancer

Magnetic resonance imaging with diffusion-weighted imaging (DWI) was found to be more accurate (91%) than CT (75%) and FDG PET/CT (71%) for peritoneal staging and to improve the quality of mesenteric/serosal metastatic spread assessment.^65^ Sensitivity of CT for nodules less than 5 mm was reported as only 11%.^66^ Average sensitivity of MRI for depicting peritoneal implants of all sizes was 84%, compared to 54% for CT, and sensitivity of gadolinium-enhanced MRI for tumors less than 1 cm was 85%-90%, compared to 22%-33% for CT.^67,68^ Accuracy of MRI (0.88) was found to be higher than CT (0.63) in determining the peritoneal cancer index (PCI) for the planning of cytoreductive surgery.^69^ Diffusion-weighted imaging and delayed gadolinium-enhanced MRI were reported as the most accurate imaging methods for detecting peritoneal tumors.^70,71^ *For assessment of peritoneal metastases*,* detailed abdominal MRI including DWI and late phase-contrast series should be preferred prior to treatment.*

#### Role of Fluorodeoxyglucose Positron Emission Tomography/Computed Tomography in Staging of Rectal Cancer

*National Comprehensive Cancer Network (NCCN) guidelines do not recommend the evaluation of patients with FDG PET/CT if contrast-enhanced CT can be performed in patients diagnosed with rectal cancer.* Fluorodeoxyglucose positron emission tomography/computed tomography can be used if contrast-enhanced CT is inconclusive or if patients have contraindications to the IV contrast agent, such as renal dysfunction or allergy. Fluorodeoxyglucose positron emission tomography/computed tomography allows whole-body staging of possible distant metastases and may clarify the diagnosis if other imaging modalities are inconclusive. Sensitivity of FDG PET/CT for characterization of pararectal lymph nodes is not high. However, distant lesions such as paraaortic, supraclavicular metastatic lymph nodes, or organ metastases can be detected in PDG PET/CT. *In patients with proven metastatic and obstructive disease*,* FDG PET/CT can be considered to detect other possible sites of metastases. Additionally, baseline imaging allows evaluation of therapy response in comparison to future studies in metastatic patients*.^59^

## Radical Surgery for Rectal Cancer

### Basic Principles

*The definitive radical surgery for rectal cancer should be total or partial mesorectal excision.* Total mesorectal excision (TME) is indicated for carcinoma of the middle and lower third of the rectum. For the oncologic principles of rectal cancer surgery, the quality of excised mesorectum is a key factor and should be complete or near-complete.^72,73^ Mobilization of the splenic flexure and ligation of inferior mesenteric vein (IMV) are crucial steps for TME. The high ligation of inferior mesenteric artery (IMA) and the ligation of IMV at the lower border of the pancreas allow tension-free anastomosis along with splenic flexure mobilization.^35,59,74^

*Positive circumferential radial margin (CRM) is one of the independent risk factors for local recurrence.^75^*

In the Dutch rectal cancer study, 267 (30%) of the patients who were treated with anterior resection (AR) with TME had an upper rectal cancer.^76-78^ In the studies reported by Sauer et al.^79,80^ the use of TME was also performed for eligible patients including those who had tumors within 16 cm from the anal verge.^81^ Total mesorectal excision was recommended for tumors at all levels. Surgeons were also encouraged to use mesorectal excision in the Medical Research Council trial.^82^ However, a partial mesorectal excision (PME) can be performed for the upper third of the rectal tumors (intraperitoneal tumors) where a distal margin is recommended to be at least 5 cm.^83,84^ It is important to recognize that distal mesorectal spread often extends further than intramural spread, with deposits found up to 3-4 cm distal to primary cancer. For the middle and lower third of the rectal cancers where TME is performed, the distal margin is recommended to be at least 1 cm.^85,86^ It has been reported that PME was a less complex procedure with a lower anastomotic leakage rate compared to TME.^87^ The morbidity rate of TME seems high with an increased risk of anastomotic leakage with low anastomoses. Up to 17% of anastomotic leakage with 11% of postoperative peritonitis has been reported after TME.^88^ *Therefore*,* the routine use of intestinal diversion has been advocated with TME.^89,90^ We routinely divert our patients as suggested.*

*The proximal distance to the tumor should be at least 10 cm. Surgical margins should be assessed together with the pathology team. Stapler doughnuts should be included for distal surgical margin evaluation.^91-93^*

The mesorectum and mesocolon should be complete for oncologic lymphadenectomy, and we recommend the high ligation of the IMA/IMV. *The number of harvested lymph nodes should be at least 12 for adequate lymphadenectomy*. Considering the fact that the high ligation of the IMA has not been shown to extend overall survival (OS), ligation can be performed by protecting the left colic artery branch by dissecting the apical lymph nodes around the origin of IMA.^35,36,59^

Intersphincteric TME can be performed for the management of the lower third of rectal cancer close to the dentate line where the anastomosis is performed at the anorectal ring or dentate level. The decision should be given preoperatively on whether to perform transanal hand-sewn anastomosis or double-stapled anastomosis.^94^ *Abdominoperineal resection (APR) should be performed after neoadjuvant treatment in patients with distal rectal tumors invading levator muscles and external anal sphincters.*

During the classical APR, surgeons preserve the levator ani, leading to dissection very close to the tumor and creating “Morson’s waist” defect. Cylindrical APR allows achieving a monobloc excision of the portion of levator muscles that are not otherwise removed during classic APR. Cylindrical APR is a better method of preventing CRM (+), particularly at the level of levator muscles.^13,94-97^

### Minimally Invasive Surgery for Rectal Cancer

*Rectal cancer surgery can be performed by preferring open, laparoscopic, or robotic approach.* Transanal TME is an emerging technique where the dissection of the mesorectum is started from the perineum at 1 cm below tumor to the all the way up. This technique can be favorable for the tumors located in the lower third of the rectum with a narrow pelvis. It is particularly preferred for intersphincteric resection.^98-101^

*Patients with cT1N0 and cT2N0 initially should proceed with surgery (TME) without chemoradiotherapy (CRT). For selected patients with mrT3a, without mesorectal fascia involvement and EMVI 0, 1, and 2, TME can be performed without receiving any neoadjuvant CRT; otherwise, neoadjuvant treatment is recommended. For patients with mrT3b, c, and d, TME should be performed following neoadjuvant CRT. Any T3 tumors located on the distal third of the rectum or close to the levator ani should receive neoadjuvant treatment before surgery. Patients should receive neoadjuvant CRT if the tumor’s distance to the mesorectal fascia is less than 1 mm.* The local recurrence rate is similar for T2 and mrT3a tumors. Compared to mrT3a, mrT3b has poor OS independent of lymph node involvement. Patients with pT3N0 and CRM (−) can be followed without adjuvant treatment after radical surgery.^36,102,103^

#### When to Perform Extended (Lateral) Lymphadenectomy?

*Lateral lymph nodes (LLNs) are defined as the lymph nodes located around the external iliac*,* obturator*,* and internal iliac vessels.* The rate of metastatic LLN for locally advanced tumor is around 5%-30%. Risk of recurrence persists even after shrinking LLN following neoadjuvant treatment. If LLN does not shrink after neoadjuvant treatment, the risk for positive lymph node is around 61%. If the lymph node is less than 5 mm, lymph node is usually negative.^103-107^ A neoadjuvant treatment is indicated for patients with clinically positive LLN on preoperative MRI staging. *Lateral lymph node dissection should be performed in conjunction with TME for patients with LLN greater than 7 mm after CRT*. The decision whether to perform LLN dissection following neoadjuvant treatment should be given at the multidisciplinary tumor meeting.^108,109^

### Management of Completely Obstructing Rectal Mass

*Diverting stoma should be created for the initial management of complete bowel obstruction in patients with extraperitoneal locally advanced rectum cancer.* *The right-sided transverse colostomy or loop ileostomy should be performed according to the surgeon’s preference.* Colostomy is the best option if the ileocecal valve is competent. Definitive stenting in rectal cancer is defined as the stenting in patients with an acute obstruction that requires preoperative decompression as a bridge to surgery with a curative intent. *Palliative stenting can be performed in patients with unresectable tumors or advanced diseases of less than 3 months of prognosis*.^110,111^ Self-expanding metal stent is not preferred as a long-term treatment due to stent migration and stent-related perforation.^112^ Self-expanding metal stent placement is not recommended for patients with extraperitoneal rectal cancer. Stenting is also contraindicated in patients with potentially curable rectal cancer.

### Basics of Pathologic Analyses for the Treatment of Rectal Cancer

*Histopathologic examination is the gold standard for the diagnosis of rectal cancer.* Obtaining the right amount of tumor tissue during endoscopy is essential for the accurate diagnosis. Although current guidelines do not specifically state the number of biopsies required, several studies demonstrated that the diagnostic sensitivity increases significantly with the increased number of biopsies taken.^113,114^ *For maximizing the accuracy of the histopathological diagnosis*,* 6 biopsies should be taken from the tumor-suspected areas.* When 6 tissue fragments were evaluated, the sensitivity of the histological examination was reported to be as high as 98%.^114^

### Assessment of Mismatch Repair Deficiency

*Immunohistochemical (IHC) testing of microsatellite instability (MSI) on tumor material, which reflects mismatch repair (MMR) deficiency*,* is recommended by the current guidelines for all CRCs*. Immunohistochemical expressions of MMR proteins (MLH1, PMS2, MSH2, and MSH6) can be evaluated both on resection and biopsy specimens.^115,116^ Interpretation may be challenging due to staining alterations on resection specimens, tissue fixation issues, and the effect of the neoadjuvant therapy. Furthermore, tumors may regress completely following neoadjuvant therapy, leaving no residual tumor material available.^117^ Tissues from endoscopic biopsy materials are reported for a reliable MMR IHC analysis. This method allows clinicians to know MMR status prior to treatment, as well as to obtain a better tissue fixation and optimal staining.^116^

When available, tumor tissues from endoscopic biopsies should be the first choice for IHC testing of MMR. If results on endoscopic biopsies are inconclusive, IHC can be repeated on resection specimens.

### Reflex Testing of Caudal-Related Homeobox Transcription Factor 2

In the literature, IHC loss of caudal-related homeobox transcription factor 2 (CDX2) in CRCs is claimed to be associated with aggressive histological features, including poor differentiation, lymph node metastasis, and lymphovascular and perineural invasion, as well as worse disease-free survival (DFS)^118^ and may benefit from adjuvant chemotherapy.^130^ Caudal-related homeobox transcription factor 2 cannot be used as independent prognostic marker; however, it is correlated with MMR status and BRAF mutation.^119^ Caudal-related homeobox transcription factor 2 immunostaining is often heterogeneous and scoring with CDX2 is not well established.

*Reflex testing of CDX2 immunostaining on rectal tumor specimens is optional due to controversies regarding its prognostic significance in the literature.* However, it can be carried out for academic purposes.

### Assessment of Malignant Polyps

Malignant polyps are defined as colorectal adenomas containing invasive adenocarcinoma that extends into the submucosa. Histologic features associated with aggressive behavior are the presence of poor histologic grade/component, positive deep resection margin, lymphatic/vascular invasion, intermediate/high tumor budding score, and invasion depth of more than 1 mm.^120,121^

*Histologic parameters including grade and type of the invasive tumor*,* tumor extension (Kikuchi levels for sessile and Haggitt levels for pedunculated polyps)*,* margin status (deep and mucosal)*,* microscopic distance from the deep margins*,* LVI*,* and tumor budding [low (Bd1)/intermediate (Bd2)/high Bd3)] should be examined and reported in the final pathology report.*

### Pathological Evaluation of Local Excision Specimens

Minimally invasive endoscopic methods are increasingly used for en-bloc resection of colorectal lesions including adenomas and early carcinomas for selected patients. These specimens should be carefully handled for gross photography and margin orientation. *Specimens should be sent en-bloc for histologic examination*. Histological parameters that should be included in the pathology report are similar to malignant polyps.

### Pathological Evaluation of Radical Resection Specimens

Appropriate handling and thorough histopathological examination of surgical specimens for rectal cancers (low AR and APR) are essential to assess the quality of the surgical treatment, as well as to predict the outcome of the patient and to determine further treatment options. Protocols of the American Joint Committee on Cancer (AJCC) and College of American Pathologists (CAP) are widely acknowledged systems to evaluate these specimens with regard to pathological staging and reporting.

Neoadjuvant chemoradiation therapy in locally advanced rectal cancer has been proven to result in significant tumor response and downstaging. *A modified Ryan scheme is recommended as a standard scoring system by the CAP to report tumor response.*

Although it is not included in the CAP and AJCC protocols, several studies have shown the prognostic significance of subdividing T3 rectal tumors according to the microscopic depth of perirectal fat invasion.^122,123^

### PAN-RAS testing

Anti-epidermal growth factor receptor (EGFR) monoclonal antibodies have been the main targeted therapies for metastatic CRCs that require knowledge of the mutational status of genes in the pathway as predictive biomarkers of response to these therapies. Epidermal growth factor receptor signaling pathways involving KRAS, NRAS, BRAF, PIK3CA, and PTEN affect response of CRC to anti-EGFR antibody therapies. According to several studies and guidelines for CAP/ASCO/AMP, KRAS and NRAS (PANRAS) mutation analysis covering second, third, and fourth exons of these genes need to be performed before starting anti-EGFR therapy. The presence of BRAF mutation (especially BRAF V600E) is not exclusive of anti-EGFR treatment choice for metastatic CRC since still there is not enough data. BRAF (V600E) mutation has prognostic importance. Therefore, KRAS, NRAS, and BRAF mutational analyses should be requested for all CRC patients before anti-EGFR treatment. KRAS, NRAS, and BRAF mutations are mutually exclusive in CRCs. Primary or metastatic CRC tissues could be used for these analyses. Pathologists must choose suitable tumor tissue which contains enough amount of invasive carcinoma cells for mutational analysis.^124-127^

*PAN-RAS (KRAS/NRAS) mutational testing is necessary for a CRC patient who is a candidate for anti-EGFR therapy. Mutational analysis should include KRAS and NRAS codons 12 and 13 of exon 2*,* 59*,* and 61 of exon 3, and 117 and 146 of exon 4* (“*expanded*”* or* “*extended*”* RAS*).* BRAF p.V600 (BRAF c.1799 [p.V600]) mutational analysis should be performed in CRC tissue in patients with colorectal carcinoma for prognostic stratification.*

### Identification of *Microsatellite Instability*-High

Tests for deficient mismatch repair (dMMR) or MSI have been recommended for all patients with CRC as a workup test for evaluating the presence of Lynch syndrome (LS). Microsatellite instability-high colorectal carcinomas have been shown to have a better overall prognosis compared with microsatellite stable tumors. Although the current gold standard for assessing tumor DNA MMR activity is a molecular MSI testing, IHC for MMR proteins has a sensitivity of more than 90% and specificity of 100%. Concordance between IHC and molecular testing is excellent. Since there are some possibilities of pitfalls for IHC-MMR, MSI molecular testing is advised as complementary. Identification of MSI-H (or MMR-deficient) CRC is important for prognosis, predictive marker of response to 5-FU-based chemotherapy, and immunotherapy. Either IHC for MMR proteins or molecular test using consensus panels can be used for detection of MSI status of tumor. *When available, tumor tissues from endoscopic biopsies should be the first choice for IHC testing of MMR.*

### Molecular Subtyping of Colorectal Cancer

During normal DNA MMR activation, MLH1 recruits its binding partner PMS2. The same combination is true for MSH2 and its binding partner MSH6. This fact is important to know while reporting MMR protein expression and directing genetic testing to the appropriate MMR gene when loss of an MMR protein expression is identified. This information is important to handle and drive workflow to identify LS/sporadic CRC carcinoma.^128,129^

The diagnosis of hereditary LS and recognition of sporadic CRCs with MSI-H have important implications regarding cancer prevention, surveillance, and management. There are several proposed algorithms for MSI testing. Most of them include IHC as prescreening, followed by confirmation with MSI by polymerase chain reaction (PCR), BRAF mutation analysis, and MLH1 methylation test. In this algorithm, loss of MSH2 and MSH6 expression by IHC indicates the presence of germline MSH2 mutation. Loss of PMS2 or MSH6 only indicates PMS2 or MSH6 germline mutation, respectively. *If the tumor shows loss of MLH1 and PMS2 expression, either BRAF mutation analyses or an MLH1 methylation test should be performed*. BRAF mutations are present almost exclusively in serrated pathway neoplasms and exclude LS. Additionally, some MSI-H tumors are BRAF wild-type. Methylation of the MLH1 gene is another reason for the loss of MLH1/PMS2 expression in sporadic CRC. Thus, if MLH1 methylation analysis shows methylator phenotype, MSI-H tumor is presumed to be sporadic and not likely a result of Lynch syndrome.^128-130^

Either MLH1 methylation analysis or BRAF p.V600 mutational analysis should be performed in MMR-deficient tumors with loss of MLH1 to evaluate for LS risk. The presence of a BRAF mutation strongly favors sporadic pathogenesis. The absence of a BRAF mutation does not exclude risk of LS. The presence of MLH1 methylation excludes the possibility of LS. Colorectal cancer is a heterogeneous disease both morphologically and at the molecular level. Lately, next generation sequencing (NGS) panels, including a large number of genes playing important role in some tumoral pathways, are giving us more information about molecular genotyping and phenotyping characterization of CRC. Comprehensive transcriptomic analysis has allowed for identification of 4 consensus molecular subtypes (CMS): CMS1 (MSI-H, 14%); CMS2 (canonical, 37%); CMS3 (metabolic, 13%); and CMS4 (mesenchymal 23%).^131-133^ Molecular subtyping of CRC is important for prognostication and determination of treatment strategies for CRC patients individually. NGS panels are getting more widely used in molecular pathology practice.

## Neoadjuvant and Adjuvant Treatment for Rectal Cancer

### Role of Radiotherapy

Both short-course radiotherapy (RT) and long-course preoperative CRT improve local control in locally advanced (cT3-4/N+) rectal cancer. *Long-course neoadjuvant treatment is usually preferred for patients with distal, T3-4, unresectable, or radiologically CRM+ tumors since it may increase the likelihood of tumor shrinkage and sphincter preservation rates.* Preoperative CRT has replaced postoperative CRT as the standard of care for locally advanced T3-4/N+ rectal cancer after the results of the German CAO/ARO/AIO-94 trial were reported.^134^ This large randomized trial showed that local control and toxicity were improved in the neoadjuvant arm. Short-course RT has also proven to improve local control, even in patients to be operated with TME in the Dutch trial.^135^ Preoperative short-course RT and long-course CRT were compared in the TROG trial and resulted in similar oncological outcomes.^136^ There was a non-significant local recurrence difference in distal tumors in favor of long-course CRT (12% vs 3%, *P* = .21). In a randomized study with unresectable cancers, long-course CRT was superior to short-course RT in terms of R0 resection, pathologic complete response (pCR) (16% vs 7%), local control (82% vs 67%), DFS, and cancer-specific survival rates.^137^

*Neoadjuvant treatment may be omitted in selected patients with T3N0, CRM*−, *and proximal rectal cancers who are thoroughly staged with MRI and to be operated with TME by an experienced team*. However, sensitivity of radiological methods still cannot provide a precise prediction for lymph node metastases. Besides, unselected patients treated with preoperative short-course RT had a lower local recurrence compared to selectively treated patients with postoperative CRT if they had circumferential margins of £1 mm (4.4% vs 10.6%) in a large randomized trial.

Early (within 1-2 weeks) or delayed surgery may be performed after short-course RT. In the initial studies with short-course RT, surgery was performed within 1-2 weeks after RT. Following trials used a delayed surgery approach (5-13 weeks after RT) in order to increase the tumor response rate. A systematic review analyzed 16 studies, in which it was shown that a lower rate of severe acute post-radiation toxicity was observed in the immediate-surgery group.^138^ However, this benefit was counterbalanced by the increase in minor postoperative complications. When the surgery was delayed, the pCR rate was about 10% higher, but R0 resection and sphincter preservation rates were similar. A pCR of over 20% was recorded after short-course RT and consolidation chemotherapy followed by delayed surgery. *Stockholm III trial randomly tested immediate surgery as a standard approach against delayed surgery (4-8 weeks) or long-course RT.^139^ Oncological outcomes were similar. However, the risk of postoperative complications was lower after short-course RT with delayed surgery.*

### When to Perform Adjuvant Radiotherapy

*Patients with pT3-4/N+ or surgical margin positive disease after surgery should receive postoperative RT*,* if not given before surgery.* Although preoperative treatment has replaced postoperative treatment as a standard of care, some patients are upstaged after surgery with a definite pathologic review of the surgical specimen. *Since the rate of local recurrence is low after a proper TME surgery for proximal T3N0 disease*, the *omission of postoperative RT may be appropriate. Selection of patients with favorable prognostic factors (<2 mm mesorectal invasion*,* grade 1-2*, *and without lymphatic or vascular invasion) for this approach may decrease the risk of local recurrence.^140^*

## Role of Chemotherapy

### Selection of Patients

Patients who will require adjuvant therapy should be considered for neoadjuvant treatment protocols. According to the NCCN guidelines, definite indications for neoadjuvant chemoradiotherapy (nCRT) are clinical T3 or T4 disease and node positivity with EUS or MRI. However, there can be discordances between the clinical and pathological staging. In a review of EUS/MRI-staged patients with clinical T3N0 tumors, 22% of mesorectal lymph node positivity was detected in resected specimens.^141^ The European Society for Medical Oncology (ESMO) guidelines are against routine delivery of preoperative RT or CRT to all patients with imaging-predicted node positivity due to the poor accuracy of categorization based on nodal size alone.^142^ The depth of extramural invasion has also been depicted as a prognostic factor. Various studies have pointed at high nodal involvement and lower survival rates for T3 tumors with >5 mm extramural invasion depth.^91,102^ Currently, TNM staging has not incorporated subclassification of T3 tumors using depth of extramural invasion; however, the ESMO guidelines suggest upfront surgery for tumors with <5 mm depth of invasion beyond muscularis propria and no threatening of levators and extranodal extension.^35^

*We recommend administering nCRT to patients who are supposed to be candidates for adjuvant CRT. Patients with cT3N0 proximal rectal cancer with >5 mm extramural invasion are also candidates for neoadjuvant CRT.*

### Type of Concurrent Chemotherapy Regimen

Both infusional 5-fluorouracil (5-FU) and capecitabine are acceptable chemotherapy regimens concurrently administered with RT. Although early trials have mainly utilized bolus 5-FU during the first and last weeks of RT, concerns of toxicity have caused the elimination of routine bolus 5-FU administration. An early trial comparing adjuvant bolus and infusional 5-FU during pelvic RT has demonstrated superior OS rates for infusional chemotherapy.^143^ However, another trial testing bolus and infusional 5-FU with concomitant radiation in the postoperative setting has yielded similar relapse-free survival and OS outcomes, with the expense of more common hematologic toxicity in the bolus 5-FU arm.^144^ Non-inferiority of capecitabine has been demonstrated in a phase III study, and local relapse and OS rates have been similar, although distant metastases were less common with capecitabine.^145^ Both infusional 5-FU and capecitabine can be used concomitantly with RT by considering their toxicity profiles.

Administration of oxaliplatin concomitantly with RT has provided a modest increase in pCR rates with clearly increased toxicity such as grade 3-4 leukopenia, diarrhea, skin toxicity, and radiation proctitis.^146-151^ There has been no DFS advantage with the addition of oxaliplatin during RT except for the German CAO/ARO/AIO-04 trial.^152^ Thus, oxaliplatin is not recommended concomitantly during RT.

*Infusional 5-FU and capecitabine can be administered concomitantly with RT. The addition of oxaliplatin to infusional 5-FU or capecitabine is not recommended due to lack of survival benefit and increased toxicity.*

### Total Neoadjuvant Therapy

Earlier delivery of full-dose systemic chemotherapy has the theoretical capacity to eradicate micrometastatic disease and decrease the risk of disease progression during treatment. Moreover, total neoadjuvant therapy (TNT) can provide an opportunity to select patients with clinical complete response (cCR) to be considered for nonoperative management (NOM). Yet, there is no phase III randomized trial comparing the standard CRT approach with TNT, and attempts to increase pCR rates have not always resulted in improved disease-related outcomes.^153^ Given the lack of strong evidence for TNT strategy, this approach still can be suggested for patients with middle or distal rectal cT4 and/or N2 tumors or those with CRM (+) T3 tumors after discussion in MDTB. Mainly, 2 pragmatic approaches have been tested to optimize the delivery of trimodality therapy: incorporation of systemic therapy before or after conventional neoadjuvant CRT. The number and type of induction chemotherapy before CRT has been variable in different studies. Induction chemotherapies have mainly included oxaliplatin either as FOLFOX or XELOX and the duration of induction chemotherapy has changed between 3 and12 weeks.^154-159^ A recent phase III trial (PRODIGE 23) has utilized the mFOLFIRINOX regimen before long-course CRT in comparison to standard long-course CRT followed by surgery and adjuvant chemotherapy. The experimental arm has yielded significantly higher rates of pCR (27.5% vs 11.5 %), DFS, and metastases-free survival. Overall survival data are not mature yet (332). Administration of chemotherapy after CRT has also been tested in several small-scale studies.^160-163^ A multi-institutional phase II randomized trial has demonstrated that increased pCR rates correlated with the number of chemotherapy cycles administered after the CRT until surgery.^164^ The most common RT regimen utilized in TNT studies has been long-course CRT. However, a phase III trial has compared the efficacy of short-course RT followed by 3 cycles of FOLFOX regimen with standard CRT protocol and interestingly has yielded superior OS outcomes without difference in DFS or local control rates. Administration of systemic chemotherapy during the “resting period” between CRT and surgery has been assessed with different strategies but none has shown survival benefit despite an increase in cCR or pCR rates.^159,164-166^ Although the method for evaluating response has differed across trials, serial digital rectal examination, rigid proctoscopy, abdominopelvic CT/MRI, and serum CEA levels can be performed before CRT when feasible or after CRT completion and at 2-3 months intervals depending on the interval between surgery and the initiation of TNT.^167^ Yet, using a variety of radiologic modalities to assess tumor regression and predicting pCR remains an area of active research due to high false-negative rates with either anatomic or functional (FDG PET/CT) imaging techniques.^168-170^

*Given the lack of strong evidence for benefit of TNT approach, it can be considered for mid or low rectal cT4 and/or N2 tumors or cT3 tumors with high risk for CRM-positivity based on MDTB decision.* Oxaliplatin-based chemotherapy, either as FOLFOX or XELOX, can be administered before or after CRT. Both induction chemotherapy followed by CRT or CRT followed by systemic chemotherapy are acceptable strategies. Completion of the planned chemotherapy during the neoadjuvant period (a total of 6 months) can be preferred due to increased compliance. Response to treatment can be done every 2 months preferentially with the tools used initially during clinical staging. Surgery can be performed 2-3 weeks after the last chemotherapy cycle or 6-8 weeks after the last RT fraction for long-course RT.

### Adjuvant Chemotherapy

The evidence regarding the efficacy of adjuvant chemotherapy/CRT for patients who have not received neoadjuvant CRT has relied mainly on early studies that utilized chemotherapy agents inferior to modern standards and a Cochrane meta-analysis which included early studies performed with 5-FU-based therapies.^171-174^ Adjuvant chemotherapy has been associated with both DFS and OS advantages. *The NCCN guidelines have offered adjuvant 5-FU concomitantly with RT and alone for pT3N0 tumors and oxaliplatin-based treatment for pT4 and node (+) disease.^59^ However*,* the ESMO guidelines have recommended a risk-adapted strategy; adjuvant chemotherapy has been suggested for only stage 3 and high-risk stage 2 disease patients such as those with unexpected adverse histopathological features; positive/close circumferential resection margin (≤1 mm)*,* perforation in the tumor area*,* pathologic T4b disease*,* an incomplete TME*,* extranodal deposits N1c*,* or nodal deposits with extracapsular spread close to the mesorectal fascia.^35^ Collectively*,* 6 months of perioperative chemotherapy can be offered including CRT either initially or after 1-2 cycles of adjuvant chemotherapy.*

Adjuvant chemotherapy with CRT can be administered to pathologic stage II and III patients who have not received nCRT. For stage II patients, infusional 5-FU alone or capecitabine is recommended. Radiotherapy can be started with the first cycle or following 1-2 cycles of chemotherapy. Although data supporting the benefit of the addition of oxaliplatin for node-positive disease in this setting are lacking, FOLFOX/XELOX regimen can be considered through extrapolation from colon cancer trials.

Selection of patients for adjuvant chemotherapy after neoadjuvant CRT is challenging since most of the trials performed among such patients have failed to demonstrate a clear survival benefit.^175-178^ Lack of standardized TME procedure, absence of observation arm in the adjuvant setting, heterogeneity of neoadjuvant treatment protocols, and failure to reach full accrual for some of the studies have limited the interpretation of results. Meta-analysis of individual patient data from four European randomized trials has also failed to demonstrate OS or DFS benefit for stage II or III rectal cancer following neoadjuvant therapy and surgical resection.^179^ However, a recent open-label phase II trial including ypStage II (ypT3T4-N0) and ypStage III (ypTanyN1-2) patients after neoadjuvant CRT has found an improvement in 3-year DFS with FOLFOX regimen compared to bolus 5-FU regimen.^180^ Similarly, the German CAO/ARO/AIO-04 trial has pointed at a DFS advantage with the addition of oxaliplatin in both neoadjuvant and adjuvant setting compared with the bolus 5-FU arm.^152^

Response to neoadjuvant therapy has not consistently been a useful tool for the selection of patients who would benefit from adjuvant chemotherapy. Unplanned subgroup analysis of some trials and nomograms has pointed at a DFS benefit for ypT3-4 or ypN2 disease (non-responders) whereas there are 2 retrospective cohort studies pointing at survival advantage with adjuvant chemotherapy for patients achieving pCR after neoadjuvant CRT.^152,180,181^ Given the lack of prospective data, recommendations should be made on individual patient basis in MDTB taking the patient-related factors into consideration.

Response to neoadjuvant therapy is not a useful tool for predicting benefit from adjuvant chemotherapy either. Each case should be discussed in MDTB, and the patient should be informed about the risks and benefits of the suggested treatment. Addition of oxaliplatin in the adjuvant setting after neoadjuvant chemotherapy may provide DFS benefit for clinical/pathologic T3-T4 and/or node-positive disease.

Currently, there are no trials addressing the optimal duration of adjuvant chemotherapy for rectal cancer. *Since the data are not conclusive*,* we recommend 4 months of adjuvant chemotherapy when neoadjuvant CRT is administered.*

## Specific Considerations

### When to Perform Surgery After Neoadjuvant Treatment

Sun et al^182^ reviewed the National Cancer Database for optimal surgical timing after neoadjuvant therapy. Eight weeks appear to be the critical threshold for optimal response. While optimal surgical timing has been previously reported to be 6-8 weeks after long-course neoadjuvant therapy and 1 week after the short course, the optimal duration of interval after CRT has been controversial.

OSTriCh^183^ group also reviewed the National Cancer Database and found that a nCRT surgery interval time of >8 weeks results in increased odds of pCR, with no evidence of associated increased surgical complications compared with an interval of 6-8 weeks. These data support the implementation of lengthened interval after nCRT to increase the chances of obtaining a pathologic complete response. In the latest analysis of 11 760 patients, the optimum interval for complete resection and downstaging was concluded as 8 weeks.^182^ Another study from Korea^184^ reported the optimal timing for curative surgery in rectal cancer when tumor response is maximal as after 7 weeks and before 10 weeks following preoperative nCRT. GRECCAR-6 randomized stage II-III patients treated with CRT into 2 groups according to the timing of the surgery as 7 or 11 weeks after completion of the CRT. Pathologic complete response was similar between groups (15% vs 17.4%), but the morbidity and complete resection rates were worse at 11 weeks.^155^

Sloothaak et al^185^ reported that delayed surgery until the 15th or 16th week after the start of CRT (10-11 weeks from end of CRT) has the highest likelihood of a pCR. Recently, waiting period was extended from 6-8 weeks to 12-16 weeks after long-course neoadjuvant therapy, waiting period was from 7 days extended to 4-8 weeks for short-course neoadjuvant therapy.

According to NCCN guidelines,^59^ surgery can be performed 5-12 weeks after long-course neoadjuvant therapy. For short-course therapy, surgery can be considered at 3-7 days or 4-8 weeks.

Another meta-analysis has recently demonstrated that pCR rates are significantly increased in patients with locally advanced rectal cancer after a waiting interval of >8 weeks after nCRT and surgery compared to a waiting interval of <8 weeks. There were no significant differences in OS, DFS, operative time, or incidence of local recurrence, postoperative complications, or sphincter-preserving surgery.^186^ *We currently recommend optimum interval time as 6-8 weeks or longer after long-course and 2-4 weeks after short-course neoadjuvant therapy for rectal cancer.*

#### Use of Chemotherapy Following Completion of Neoadjuvant Treatment Until Surgery

Different CRT regimens with consolidation chemotherapy may lead to increased rates of complete regression. Most of the reduction in tumor metabolism after long-course neoadjuvant CRT occurs within the first 6 weeks from RT and reaches the maximum effect at the 10th week. Patients undergoing CRT with consolidation chemotherapy tumors are less likely to regain metabolic activity within 6-12 weeks.^165,187^ The other issue for nCRT is that systemic recurrence remained unchanged despite nCRT. The only significant prognostic factor was pathologic complete response after CRT. Consolidation chemotherapy is adding several cycles of chemotherapy between nCRT and surgery. It could increase pathologic complete response and could lead to better oncologic outcomes. The Konclude trial reported that consolidation chemotherapy showed better pathologic complete response rates and 3-year DFS than adjuvant chemotherapy alone in the patients who received nCRT and adjuvant chemotherapy alone.^188,189^ Recently, Marco et al^190^ reported that adding modified Folfox 6 after CRT and before mesorectal excision increases compliance with systemic chemotherapy and DFS in patients with locally advanced rectal cancer. *In conclusion*,* consolidation chemotherapy increases pathologic complete response rates. However*,* specific selection criteria are not well defined yet. The decision for applying consolidation chemotherapy should be decided at the MDTB on an individual basis. Chemotherapy should be stopped 2 weeks prior to surgery.*

#### What is the Best Imaging Modality to Evaluate Tumor Response After Neoadjuvant Therapy?

Proper evaluation of tumor response to nCRT in locally advanced rectal tumors plays an important role in determining the treatment and type of the surgical method.^191^ While it is optional in some guidelines,^27,192^ the value of restaging MRI has been pointed out by many authors.^26,193^ The accuracy of MRI decreases after nCRT due to fibrosis, wall thickening, and inflammatory changes.^194^ The reported accuracy rates of post-CRT MRI for T staging and N staging were 48% and 63.8%, respectively.^194^ In a large meta-analysis, it was reported that the mean sensitivity rate of MRI for T staging increased from 50.4% to 73.6% with the addition of DWI after CRT.^195^ Although abdominal/pelvic CT after neoadjuvant therapy has been shown to identify resectable liver metastases in only 2.2% of patients (95% CI, 0.8%-5.1%),^196^ chest and abdominal imaging have still been recommended for the assessment of distant disease.^59^ According to the NCCN guideline,^59^ re-staging should be the same as pretreatment evaluation with chest CT, abdominal CT or MRI, and rectum-specific pelvic MRI.* For the evaluation of response after nCRT, we recommend high-resolution rectal MRI including detailed diffusion imaging for local staging. This can be extended as an abdominal MRI to assess the probable remote intraabdominal disease. Fluorodeoxyglucose positron emission tomography/computed tomography is not routinely indicated but can be performed based on the assessment of the risk factors on an individual basis. An endoscopic evaluation should be done prior to surgery.*

#### What is the Optimum Time for Ileostomy Closure?

There is no consensus regarding the best timing for temporary stoma closure after proctectomy for rectal cancer, especially when patients require adjuvant chemotherapy. Figueiredo et al^197^ suggested that the timing of temporary stoma closure can influence postoperative morbidity. They concluded that the best results of stoma closure were obtained within 90 days after radical surgery. Early closure of the temporary ileostomy could reduce complications for rectal cancer patients. Danielsen et al^198^ reported that early closure of temporary stoma is safe even 8-13 days after rectal resections. There are many additional data available regarding safe early stoma closure.^199^

The CLOSE-IT study is looking for optimal ileostomy closure timing, and the study is still recruiting patients.^200^ *At the moment*,* there is no strict rule for ileostomy closure timing. In our practice*,* delayed closure is routinely performed.*

#### Nonoperative Management of Rectal Cancer

A watch-and-wait approach for patients with a cCR to neoadjuvant chemoradiation could avoid the morbidity of conventional surgery for rectal cancer. However, the safety of this approach is unclear.^201^

In 2004, Habr-Gama et al^157^ compared the outcomes of 71 patients who were observed without surgery following complete clinical response who had incomplete clinical responses but complete pathologic responses post TME. The OS and DSF rates at 5 years were 100% and 92%, respectively, in the nonoperative group compared to 88% and 83%, respectively, in the resected group. However, other studies did not achieve such impressive results, and many clinicians were skeptical of this approach.^202^ Several systematic reviews have been published on the nonoperative approach.^201,203-205^ They all show that the approach is likely safe with the use of resection in patients with tumor regrowth, but that the data are very limited.

Despite the impressive results of prospective trials, many still believe that longer follow-up, larger sample sizes, and additional careful observational studies are needed before patients with cCR are routinely managed by the watch-and-wait approach.^205^ NCCN guideline panel believes that NOM and the proper approach for patients who are unfit for surgery and/or desire a stoma-free treatment may be considered in centers with experienced multidisciplinary teams after a careful discussion of the patients’ risk tolerance.

There are some problems regarding the decision method of cCR. Recent studies have found that neither FDG PET/CT nor MRI or CT can accurately determine a pathologic complete response, which makes it difficult to select appropriate patients for NOM.^59^

*In conclusion, the current evidence cannot support routine use of NOM in clinical practice. Per NCCN recommendation*,* patients who are unfit for surgery and/or desire a stoma-free treatment may be considered for NOM in centers with experienced multidisciplinary teams after a careful discussion of the patients*’* risk tolerance. This approach can be used in clinical trials or after thorough counseling with the patient on the outcomes of all treatment options.^206^*

#### Metastatic and Recurrent Disease

Patients with metastatic rectal cancer can present in 3 clinical scenarios: upfront resectable, potentially resectable, and nonresectable disease.

The majority of the patients with metastatic rectal cancer (70%) are nonresectable at presentation.^207^ Thus, the goal of the treatment is to prolong survival and increase the quality of life. A subset of patients with metastatic rectal cancer (30%) present with an oligometastatic disease in the liver and/or lung, local recurrence after definitive treatment, or limited intraabdominal disease.^207^ In these patients, there is a chance for curative surgical treatment. One-third of these patients are upfront resectable and two-thirds of them are potentially resectable after conversion therapy with systemic and/or local treatments. The probability of downstaging a patient with unresectable disease to resectable disease is 10%-20%.

In large series, it was seen that 77% of the patients with metastatic colorectal cancer had unresectable liver metastases at the time of diagnosis,^207^ and 13% of the patients with unresectable liver metastases were significantly downstaged with conversion therapies. This means that 33% of metastatic CRC with liver metastases can be resected up front or after conversion therapy.^207^ The survival of patients who are resected following conversion chemotherapy is similar to that of patients whose diseases are resectable at diagnosis.^208^

If both the primary tumor and metastases are resectable at diagnosis, one approach is to start with short-course pelvic RT followed by synchronous resection of the primary and metastatic disease. However, there are other approaches to integrate systemic chemotherapy into preoperative treatment, rather than postponing to the postoperative period.^35,59^ In all of these approaches, pelvic RT should be completed before surgery. Short-course RT is preferred over long-course CRT.^35^ The alternative approaches are as follows: (i) initial chemotherapy followed by short-course RT and surgery, and (ii) short-course RT followed by chemotherapy followed by surgery.^35,59^ *Starting chemotherapy before surgery is a more widely preferred approach because chemosensitivity and natural course of the tumor can be determined. There is no consensus on the best approach for resection of the metastases and primary tumor. It is recommended to be determined by MDTB. Resection can be either synchronous or in a staged fashion. This decision depends on the preference of the surgeon*,* extent of resection*,* and general condition of the patient.*

#### Management of Rectal Cancer with Potentially Resectable Liver Metastases

*Rectal cancer patients with potentially resectable liver metastases have a chance to be cured surgically if the response to conversion chemotherapy is sufficient.* The resection rate of metastases is associated with the objective response rate.^209^ Therefore, in rectal cancer with synchronous potentially resectable liver metastases, systemic treatment with the highest response rate should be selected depending on the molecular characteristics of the tumor.

The benefit of pelvic RT in these patients is unclear as there are no randomized trials. In 2 retrospective studies, patients who did or did not receive RT had similar rates of local and OS.^210,211^ The recurrences usually involved distant sites rather than locoregional recurrences, even in patients treated without pelvic RT.^211^ Although, the benefit of RT on OS has not been established, prevention of local recurrence through the addition of pelvic RT is an important goal considering the morbidity of locoregional recurrence. Thus, the efforts should focus on achieving margin-negative resections at the earliest moment, while not allowing delays in systemic treatment and avoiding locoregional recurrences. Although consensus guideline from the NCCN suggests both short-course RT and long-course CRT, we prefer short-course RT which is also supported by the ESMO.^35,59^

*One of the following strategies is acceptable in rectal cancer with potentially resectable liver metastases: (i) initial chemotherapy followed by RT then resection (synchronous or staged)*,* and (ii) initial RT followed by chemotherapy and then resection. It is recommended to be determined by MDTB in an individual manner*.

#### Role of Intraoperative Ultrasonography

Intraoperative ultrasound (IOUS) is recommended as a standard modality due to its superiorities for detecting unrecognized liver metastases with conventional imaging modalities.^212,213^ Intraoperative ultrasound is a useful tool to confirm tumor location during the operative period, identify the resection margins, and facilitate parenchymal transection.^214^ In a systematic review, IOUS and laparoscopic ultrasonography (LUS) performance for detecting synchronous liver metastases in patients undergoing primary colorectal carcinoma surgery was evaluated. It was reported that the detection rate of additional liver metastasis was ranging between 32% and 57% in patients who had IOUS and 2%-13% in patients who had LUS compared to preoperative contrast-enhanced CT and/or MRI.^215^ *Therefore*,* we recommend an evaluation with IOUS in patients undergoing surgery for metastatic liver disease, regardless of the type of surgery (open or laparoscopic).*

Assessment of Resectability and Principles of Surgery for Hepatic Metastases

The term “*resectability*” means more than just “feasibility of surgical removal” in practice; in fact, it also covers oncological reasoning and patient selection. From a technical perspective, 2 criteria must be fulfilled to accept liver metastases as “*resectable*”: (1) R_0_-resection should be possible, and (2) future liver remnant should be sufficient. If one of these criteria is not fulfilled, then the term “*potentially (borderline) resectable*” is used. And if none of these criteria is fulfilled, then liver metastases are considered “*unresectable*”.^35,59,216,217^

A multidisciplinary meeting discussion, in which an experienced hepatobiliary surgery team is involved, is the best way to assess resectability by all means and determine an individualized treatment algorithm for each patient. The assessment of resectability is of paramount importance because the major determinant of survival is metastatic disease in stage IV rectal cancer, and surgical resection is the only potential curative treatment for liver metastases. *Liver resection is the gold standard treatment for liver metastases in rectal cancer patients since the best oncologic outcomes are achieved with the R_0_-resection of metastatic disease.^35,59,216^ Thus*,* all rectal cancer patients with resectable liver metastases should be considered potential candidates for liver resection. The timing and technical aspects of liver resection are best determined by a multidisciplinary approach.* Debulking surgery and palliative surgical procedures for liver metastases have no positive impact on oncologic outcomes in rectal cancer patients.^35,59,216^ Moreover, they can decrease the quality of life and survival by surgical complications and lead to a significant delay in systemic treatment. Thus, resectability should be cautiously assessed before and during the surgical procedure to avoid futile liver surgery. It is extremely difficult to analyze the impact of surgical margin status on oncologic outcomes in liver resection for liver metastases of rectal cancer because of the independent variables such as systemic therapy, tumor burden, and genetic mutations.^217-221^ Nevertheless, best oncologic outcomes are achieved with R_0_-resection, and therefore, it should always be the aim of surgical treatment. However, it should also be emphasized that the risk of R_1_-resection should not preclude liver resection.

Both the definition of R_0_-resection and the optimal width of surgical margin are still a matter of debate.^222^ In a recent meta-analysis of 34 retrospective studies, the oncologic outcomes were found to be superior with ≥10 mm clear surgical margins when compared to those with <10 mm.^223^ However, there are also numerous studies reporting that there is no significant difference in oncologic outcomes with any width of clear surgical margins.^224-228^ *According to current evidence*,* achieving a clear surgical margin of ≥10 mm should be the aim, but ≥1 mm can be accepted to be adequate.*

*When the tumor is exposed during resection*,* we recommend extending the resection margins.^227-229^* The influence of the utilization of the frozen section of the specimen and re-resection to obtain clear margins when the frozen section reveals R_1_-resection on oncologic outcomes is unclear.^229-230^ Thus, it is up to the surgeon’s discretion to perform a frozen section procedure. It can be impossible to get clear surgical margins in tumors adherent to major vascular structures that cannot be sacrificed. Such tumors can be removed by separating them from the vessel, which is called a “*vascular*” R_1_-resection. Several studies reported that the oncologic outcomes of vascular R_1_-resection are similar to that of R_0_-resection.^231^

#### Surgical Technique for Liver Metastases of Rectal Cancer

Parenchyma-sparing and anatomic resections have similar oncologic outcomes, and therefore, *parenchyma-sparing* (non-anatomic, irregular, and atypical) liver resections should be preferred over anatomic resections in liver metastases, if possible.^232-234^

The studies comparing the surgical and oncologic outcomes of open and minimally invasive liver resections failed to show any statistical difference.^235-238^ Although minimally invasive procedures have some advantages, such as enhanced recovery and reduced blood loss, they demand a high level of experience and technical skill.

In fact, so-called “liver-first” approach is usually not a “true” liver-first because liver surgery is performed after systemic treatment, and it is indeed a “chemo-first” approach. *We suggest that up-front liver resection or* “*true*” *liver-first approach may be an option in patients with solitary, small (≤3 cm) metastases if the metastasis is likely to disappear during or after systemic therapy and can be resected easily with low morbidity. Optimal surgical sequencing is yet to be defined; however, liver-first, primary-first, and simultaneous resection after systemic treatment have similar oncologic outcomes, and therefore, each approach may be considered in individualized treatment protocols.^239^* *Owing to high morbidity and mortality rates*,* we recommend considering a staged procedure if a major liver resection is required.^240^ Otherwise*, *simultaneous resection may be a viable option.*

In the United States, systemic therapy is considered the initial step in the management of patients with metastatic rectal cancer regardless of resectability.^59^ In contrast, the guidelines of some European and Eastern countries recommend up-front surgery for metastatic rectal cancer if the primary and metastases are apparently resectable.^35,216^ Since there is yet no high-level evidence to support any of these approaches, both up-front surgery (primary-first, liver-first, or simultaneous resection) and systemic therapy may be the initial step in the management of rectal cancer patients with liver metastases if the whole tumor burden is clearly resectable.

There is yet no randomized study comparing surgical resection with other locoregional therapies in resectable liver metastases of rectal cancer. Since liver resection is currently considered the gold standard treatment, non-surgical locoregional therapies should be used as an alternative to surgery or can be combined with surgery only in individualized treatment protocols.

Patients with a solitary, small (*≤2 cm*), centrally located metastasis that can safely be removed only by major liver resection, particularly with right hepatectomy, may be treated by ablative procedures. In addition, surgical resection can be combined with ablative procedures in patients with multiple, bilobar metastases in whom there are concerns about the quality and quantity of future liver remnant to clear the liver from all macroscopic lesions. Systemic treatment has the potential to convert initially unresectable or borderline resectable metastases to resectable ones. Moreover, systemic therapy provides a clear survival advantage even in patients in whom resectability cannot be achieved by any means. The next step after systemic therapy should be determined up to the objective response. Patients with chemo-sensitive tumors should be re-evaluated for resectability. Otherwise, it is advised to continue with second-line chemotherapy.^35,59,216^

##### Technical Maneuvers to Enhance Resectability in Rectal Cancer Patients with Synchronous Liver Metastases

Even if an objective response to systemic therapy is achieved, initially potentially resectable and unresectable liver metastases may not be converted to resectable metastases. As mentioned above, the obstacle to resectability can be either low likelihood of achieving R_0_-resection or inadequate future liver remnant or both. In this setting, certain technical maneuvers are currently available to increase or provide resectability. Selective internal radiotherapy (SIRT) and ablative procedures can shrink or destroy the tumors and thereby increase the resectability rates. Moreover, SIRT has been shown to induce contralateral liver hypertrophy, if not as much as portal vein occlusion (PVO) does.^241^ If the concern is the sufficiency of the future liver remnant, then PVO is the best option.^242^ Furthermore, combined utilization of aforementioned maneuvers with different surgical techniques such as 2-stage liver resection with or without PVO, the combination of SIRT or ablative procedures with PVO, and associating liver partition and portal vein ligation for staged hepatectomy(ALPPS) can be used to manage with concerns about both R_0_-resection rate and future liver remnant.^242-245^ *Briefly*, *technical maneuvers to increase resectability are justified in rectal cancer patients with synchronous liver metastases that have responded to systemic treatment but are still potentially resectable or unresectable.*

#### Timing of Liver Resection

Some authors suggested that disease progression should not be considered an absolute contraindication for liver resection unless liver metastases have become unresectable.^246^ *This may particularly be true for patients whose metastatic tumors remain stable after systemic therapy has been completed.* Perioperative chemotherapy may increase DFS but has no impact on OS with the exception of chemo-naive patients.^59,247^

Radiologic and metabolic response to chemotherapy may not correlate with pathologic response in liver metastases.^248,249^ Risk of recurrence exits in patients with complete radiologic and/or metabolic responce to neoadjuvant therapy due to the possibility of viable cancer cells.^249^ *Thus*,* surgical resection of metastatic tumors with curative intent should be considered for definitive treatment.^248,249^*

Patients with metastatic rectal cancer who have diagnosed to have an unresectable disease may become resectable during the systemic treatment course. R_0_-resection in patients who have initially resectable disease and those who have had an initially unresectable disease and have resectable disease after conversion chemotherapy are similar.^250,251^ *Therefore*,* all patients who become resectable following systemic therapy should be reevaluated for surgical resection.*

#### Management of Oligometastatic and Polymetastatic Rectal Cancer

*While up-front surgery in patients who have resectable lung or liver metastasis can be performed*,* it is widely accepted that the initial step in the management of such patients should be the control of systemic disease with chemotherapy*. It is generally preferred to perform staged procedures because of the high morbidity and mortality rates associated with multiorgan resections; however, simultaneous resection may also be an option in a highly selected subgroup of patients.^35,59,216^ Surgery for rectal primary tumor should be avoided as much as possible unless there is an obstruction or perforation. In those patients with nearly obstructing lesions, short-course RT or insertion of stents may allow avoiding surgery. If these approaches do not result in palliation of the symptoms or prevent complete bowel obstruction, a diverting stoma or palliative resection can be performed.

#### Management of Isolated Lung Metastases

Overall, the 5-year survival rate has been reported to reach nearly 70% in patients with pulmonary metastases undergoing metastasectomy.^252^ In patients with recurrent isolated pulmonary metastases, repeated resections can be offered selectively to improve long-term survival.^253^ The presence of synchronous or metachronous liver metastases is not a contraindication for pulmonary metastasectomy if complete resection of all sites of disease is possible. *Surgical resection may result in significant survival advantage in rectal cancer patients with isolated synchronous*,* metachronous*, *or recurrent resectable lung metastases.^254-257^ The logic behind the management of isolated lung metastases should be similar to isolated liver metastases.*

## Standards for the Treatment of Loco-Regional Recurrences

*The gold standard treatment to obtain the longest survival for patients with loco-regional recurrence is R0 surgical resection.* The presence of multiple distant metastases, local resectability, and prior treatment modality are the factors that can be considered in the management of loco-regional recurrence of rectal cancer.^258^ The NCCN guideline recommends surgery for isolated pelvic and anastomotic recurrences and chemo and/or RT for unresectable disease. Debulking is not an option for recurrent rectal cancer.^35,59^ *Optimal interval is between 8 and 12 weeks between resection of the primary and metastatic lesion. The resectability of metastatic lesions should be evaluated every 2 months.^259,260^*

Management strategies should be calibrated under the supervision of an MDTB for the management of loco-regional recurrences with radical surgery and hyperthermic intraabdominal chemotherapy. Feasibility of an R0 resection, benefit of up-front chemo-radio treatment, and use of ablation for distant metastases which are not candidates for resection are the denominators of radical surgery for local recurrences.^258,261^ While distant organ metastases, paraaortic and supradiaphragmatic involved lymph nodes, S1-S2 invasion are the relative contraindications for pelvic exenteration, lumbar vertebral invasion and being unfit for major surgery are the exact contraindications.

The role of hyperthermic intraperitoneal chemotherapy (HIPEC) with radical surgery for advanced rectal cancer is still under investigation. In the 4 randomized controlled trials conducted in patients with peritoneal dissemination, mitomycin-C, 5-FU, and oxaliplatin were used for HIPEC.^262-265^ As there is no randomized clinical trial comparing these agents, any of them can be used in patients undergoing cytoreductive surgery and HIPEC. *It can be a treatment option in patients with perforated tumor*,* peritoneal carcinomatosis without extraperitoneal metastases. However*, *this treatment should be planned with an MDTB considering requirement of a major liver resection and the PCI.*

### Is There a Role of Radiotherapy in Locoregional Recurrent Disease?

*Re-irradiation of rectal cancer could be an option in selected patients to support resectability of tumor and long-term survival.* The results of 375 patients who re-irradiated for rectal cancer had a median survival of 39-60 months following radical surgery with a good symptomatic relief.^266^ Reirradiation was mostly administered using hyperfractionated (1.2-1.5 Gy twice daily) regimens or 1.8 Gy once-daily schema with concurrent chemotherapy to a median total dose of 30-40 Gy; With inconsistent target definition, mostly the gross tumor volume (GTV) with 2-4 cm margins, acute toxicity may develop in 9%-20% of patients^267^ and associated with diarrhea. The hyperfractionated CRT schema should be preferred to limit late toxicity.^266^

### Choice of Chemotherapy for Metastatic Rectal Cancer

*The active agents in treatment of metastatic rectal cancer are 5-FU*,* oxaliplatin*,* irinotecan, capecitabine*, *bevacizumab*, *aflibercept*,* cetuximab and panitumumab, regorafenib*,* and trifluridine-tipiracil (TAS-102).*

#### Which Molecular Tests Should Be Routinely Analyzed in Clinical Setting for Managing Metastatic Disease?

There are several molecular markers used in CRC as prognostic and predictive factors. *Mismatch repair deficiency status testing*,* extended RAS* (*KRAS and NRAS*) *mutational analysis*, BRAF* V600 mutational analysis*,* and HER2 amplification* *should routinely be ordered*.^127^

RAS mutations predict the efficacy of anti-EGFR agents. *BRAF* mutations have both prognostic and predictive significance. The evidence is not sufficient to recommend the use of *BRAF* mutational status as a predictive biomarker for response to anti-EGFR inhibitors. There are inconsistent results from 2 meta-analyses addressing the benefit of anti-EGFR therapy in patients with *RAS* wild-type but *BRAF*-mutant CRC.^268,269^

#### Choice of Chemotherapy in Patients with Up-Front Resectable Liver Metastases

There are several options to be used in patients with upfront resectable liver metastases.^59^ *The appropriate regimens are FOLFOX* (*oxaliplatin plus LV and infusional FU*),* XELOX* (*capecitabine and oxaliplatin*), *FOLFIRI* (irinotecan* plus LV and infusional FU*),* FOLFOXIRI* (*infusional FU*,* LV*, *oxaliplatin*,* and irinotecan*). Oxaliplatin-based regimens are more widely used in this setting; however, for patients who had already received adjuvant FOLFOX, FOLFIRI is a good option.

#### Choice of Chemotherapy in Patients with Potentially Resectable Liver Metastases

A regimen with a high rate of objective response is typically chosen to increase the chance of resection. Any of the following regimens can be used: FOLFOX, XELOX, FOLFIRI, and FOLFOXIRI. Oxaliplatin- or irinotecan-based doublet therapies have similar efficacy.^270-272^ *The choice of regimen depends on the toxicity profile or prior exposure to adjuvant chemotherapy. FOLFOX or FOLFOXIRI are usually the preferred doublet regimens at our institutions.*

### Choice of Chemotherapy in Patients with Non-resectable Disease

*Oxaliplatin-based* (*FOLFOX and XELOX) or irinotecan-based chemotherapies* (*FOLFIRI*)* or triplet combination* (*FOLFOXIRI*)* are used as first- and second-line therapies.* The best way to combine and sequence these agents is still not established. *The choice of regimen depends on prior exposure to chemotherapy*,* comorbidities of the patient*,* and the patient’s and the physician’s choice.* Access to all active agents is more important than a particular treatment sequence of specific regimens. Each chemotherapy/biologic treatment line is associated with longer survival.^273,274^ Regorafenib and trifluridine-tipiracil (TAS-102) are used in the treatment of patients with metastatic CRC who have been previously treated with fluoropyrimidine-, oxaliplatin-, and irinotecan-based chemotherapy and biologic agents.

### Duration of Preoperative Chemotherapy

The risk of chemotherapy-related liver toxicities depends on duration of preoperative therapy and the interval between last chemotherapy and surgery.^275,276^ *Limiting preoperative chemotherapy to 8 cycles (16 weeks) decreases the risk of chemotherapy-associated liver injury and avoids postoperative complications without any decrease in pathologic response rate*.^4^ If the interval between the last chemotherapy and resection is 4 or fewer weeks, the patients are more likely predisposed to postsurgical complications.^276^

### Choice of Biological Agent

There are several selection criteria for the biological agents: biomarker analysis, location of the primary tumor, intent of the therapy (curative vs palliative), and co-morbidities of the patient. The biological agents used in metastatic CRC targets are either angiogenesis or the EGFR. *The agents targeting angiogenesis pathway are bevacizumab*, *aflibercept*,* and regorafenib. Bevacizumab is the only antiangiogenic agent approved in first-line treatment. Aflibercept can be used in second-line treatment in combination with FOLFIRI. Regorafenib can be used after second-line treatment in patients who have been previously treated with fluoropyrimidine-, oxaliplatin-, and irinotecan-based chemotherapy, an anti-vascular endothelial growth factor (VEGF) agent*,* and anti-EGFR therapy (if* RAS *wild type). Two monoclonal antibodies targeting the EGFR are* cetuximab* and* panitumumab.

Cetuximab and panitumumab are only effective in the patients whose tumors have wild-type *RAS* (*NRAS*, *KRAS*) oncogene. In addition to the RAS mutational status, the location of the primary tumor is another factor influencing the efficacy of anti-EGFR agents.^277^ In left-sided CRC, as in rectal cancer, without RAS and BRAF mutation, an anti-EGFR-containing regimen is preferred as a first-line treatment, because OS is superior with anti-EGFR-containing regimens when compared with bevacizumab-containing regimens (med. OS 39 mo vs 33 mo).^277^ If FOLFOXIRI is chosen for first-line therapy, bevacizumab or anti-EGFR therapies (cetuximab or panitumumab) can be combined with chemotherapy depending on the RAS and BRAF status.^278-281^ FOLFOXIRI and bevacizumab combination is associated with a significant overall response rate, leading to a probability of surgical conversion of distant metastases approaching 40%, with 28% of patients having an R0 resection.^279^ Similarly, 2 phase II trials combining either cetuximab or panitumumab with FOLFOXIRI showed high response rates, high probability of conversion rates, and R0 resection in RAS-wild and BRAF-wild patients.^280,282^

In tumors with BRAF V600E mutation and wild-type RAS, response to anti-EGFR agents is unlikely.^268,269^ *In RAS mutant or BRAF mutant disease with potentially resectable liver metastases, triplet chemotherapy* (*FOLFOXIRI*)* with or without bevacizumab is preferred. However*,* if the patient is not fit enough for triplet chemotherapy*,* a doublet chemotherapy regimen with or without bevacizumab is an alternative option.*

*In patients with BRAF V600E mutation and unresectable metastases who have already received at least 1 line of chemotherapy*, *resistance to EGFR-targeted agents may be overcome with concurrent use of BRAF inhibitors like vemurafenib; however, the data are still limited.^283^*

#### Is There a Role of Using Biological Agents in Up-Front Resectable Patients?

Combining bevacizumab with chemotherapy in patients with up-front resectable CRC results in marginal benefits and risk of major complications.^284^ *Addition of anti-EGFR agents to up-front FOLFOX, even in RAS wild-type patients, results in worse progression-free survival.^285^ We do not recommend use of bevacizumab or anti-EGFR agents in this setting.*

### Benefit of Postresection Adjuvant Therapy

Adjuvant systemic chemotherapy is usually recommended in patients who have undergone metastasectomy of hepatic and/or pulmonary metastases, although there is not enough evidence from clinical trials demonstrating a survival benefit.^286,287^ The most commonly preferred regimen is a oxaliplatin-based chemotherapy regimen like FOLFOX.

*In patients with up-front resectable metastases*,* the addition of bevacizumab or cetuximab to an oxaliplatin-based chemotherapy regimen is not recommended after resection of hepatic or pulmonary metastases. In patients with potentially resectable metastatic CRC*, the *addition of a biologic agent can be planned in the perioperative setting*.^288^

### Role of Immunotherapy in Rectal Cancer

The benefit of immunotherapy with PD-1 inhibitors is limited to the subset of tumors with high levels of MSI-H/dMMR. Tumors with MSI-H or with MMR genes are susceptible to immune checkpoint inhibitors. *Pembrolizumab and nivolumab are 2 immune checkpoint inhibitors that have been shown to be effective in patients with MSI-H or dMMR metastatic CRC that has progressed following conventional chemotherapies.^289,290^*

The percentage of MSI-H/dMMR stage IV colorectal tumors ranges between 3.5% and 6.5%.^291-293^ Incidence of MSI-H/dMMR tumors is the lowest in rectal cancers when compared with the other parts of the colon.^292^

### Preoperative or Adjuvant Radiotherapy for Metastatic Rectum Cancer

Kim et al^294^ performed a propensity score-matched analysis and meta-analysis of published literature of patients with stage IV rectal cancer who underwent TME between August 2001 and December 2011 to evaluate the impact of RT on oncologic outcomes. Two groups of 39 patients each were stratified based on patients receiving adjuvant pelvic RT (RT group) and those who did not (non-RT group) using their propensity scores. The local recurrence-free survival (LRFS) of the RT group was significantly higher than that of the non-RT group (2-year LRFS: 100% vs 83.6%, respectively, *P*  = .038), while the overall DFA and distant metastasis-free survival rates were similar for both groups; adjuvant pelvic RT was highlighted to improve loco-regional control in patients with stage IV rectal cancer eligible for TME.^294^

As the phase-III trial (CAO/ARO/AIO-94) for 4 non-stage patients by Sauer et al^79^ encouraged the preoperative/neoadjuvant CRT approach in comparison to postoperative CRT due to improved local control, acute, and long-term side effects, the neoadjuvant approach also in metastatic patients sounds reasonable. Agas et al^295^ published the only meta-analysis in the neoadjuvant RT setting of metastatic rectum cancer covering 8 studies (1 CRT, 5 retrospective cohorts, and 2 population-based studies). Patients receiving neoadjuvant RT were documented to have a decreased rate of local recurrence at 2 and 5 years compared to no RT; the review demonstrated a significant difference in 2 and 5 years (neoadjuvant RT vs no RT: 10.1% vs 23.8% and 15.9% vs 26.9%, respectively). Pooled analysis from 5 retrospective studies also revealed significantly improved LRFS with neoadjuvant RT (risk ratio [RR] 1.15; 95% CI: 1.01-1.31, *P* = .03), which was maintained in the subgroup who had metastasectomy (RR 1.18; 95% CI: 1.01-1.37, *P* = .04). Moreover, statistically significant benefit with neoadjuvant RT continued in 5-year OS (RR 1.47; 95% CI: 1.14-1.89, *P* = .003) but not in the subgroup which underwent metastasectomy (RR 1.31; 95% CI: 0.94-1.82, *P* = .11).

*Based on these results in patients with metastatic rectum cancer*,* neoadjuvant RT was advocated especially in patients with following features: young age*,* low lying tumor*, *T4 lesion*,* who underwent metastasectomy*,* and who received chemotherapy containing oxaliplatin*.^295^ On the other hand, a retrospective analysis by Lin et al^296^ in 297 consecutive patients diagnosed with stage IV rectum cancer with synchronous metastasis demonstrated that younger age (hazard ratio (HR) = 0.662, *P* = .016), lower CEA level (≤20 ng/mL) (HR = 0.531, *P* = .001), no metastasectomy (HR = 3.214, *P* < .001), and no CRT (HR = 1.844, *P* = .019) were independent prognostic factors after controlling for other confounding factors, where the survival benefit of CRT was restricted only to patients who underwent subsequent metastasectomy.

#### Which Radiotherapy Schedule Is Preferred, Short or Long?

*NCCN divides M1 rectum cancer patients into 2 categories based on CRM. If the margin is less than 1 mm*,* the treatment pathway starts with any treatment containing some form of RT*, *in contrast*,* if the CRM is clear, treatment starts with either FOLFOX*,* CAPEOX*, *5FU/LV*,* or capecitabine regimen followed by RT before surgery.* There are no certain parameters to address short- or long-course RT schema.^59^

In a Dutch phase-II trial conducted between 2006 and 2010, Bisschop et al^297^ reported neoadjuvant short-course RT followed by systemic therapy with capecitabine, oxaliplatin, and bevacizumab and subsequent radical surgical treatment of primary tumor and metastatic sites. The long-term results of 50 patients after a minimum follow-up of 6 years with a median follow-up time of 8.1 years (range: 6.0-9.8) displayed 16 patients (32.0%) being alive, and of which, 14 (28%) patients were disease-free. The median OS was 3.8 years (range: 0.5-9.4), and out of 36 patients who could receive radical treatment, 2 (5.6%) had local and 29 (80.6%) had distant recurrences. Having pCR was statistically significant in median recurrence-free survival (pCR vs non-pCR: 16.2 vs 6.6 months, log-rank test, *P* = .039).^297^

*The most validated short-term RT schema has been 25 Gy in 5 fractions*,* and immediate surgery in 7-10 days following RT is recommended if CRM is clear and there is no need for regression. However, if regression is required, such as with close/positive CRM*, *tumors located at lower 1/3*,* and poor response to chemotherapy*,* long-course RT concurrent with chemotherapy could be encouraged to enhance local response*,* along with more time to increase regression.*

#### What is the Role of Brachytherapy?

*The role of high-dose-rate (HDR) brachytherapy (BT) in the management of operable rectal cancer is not well defined,^298^ while most common indications for BT were mainly cT3N+ tumors and tumors <10 cm from the anal verge*, *as well as small numbers of T2*, *T4*,* and N0 tumors included in some studies.* In a systemic review consisting of 22 studies, preoperative HDR BT with CRT provided a pCR rate ranging between 18% and 31% and sphincter preservation rate ranging between 29% and 57%, and preoperative BT alone demonstrated pCR rate ranging between 10.4% and 27% (weighted-mean 23.8%), R0 rate of 96.5% (1 study), and sphincter preservation rate of 53.8%-75.8% (weighted-mean 59.4%). These results confirmed that preoperative HDR BT either alone or in combination with CRT may result in a better pCR but not necessarily translate into a better survival in comparison to outcomes with preoperative CRT.^298^

When HDR BT was combined with CRT, the HDR BT was either delivered as 5 Gy or 10 Gy in 1 fraction or as 10 Gy in 2 fractions. Brachytherapy was prescribed at 10 mm from the applicator surface. When HDR BT was prescribed alone, usually 26 Gy delivered in 4 fractions was the most preferred dose to the clinical target volume defined as GTV and intra-mesorectal extension seen on MRI.^298^ *Overall, HDR BT is not recommended in routine practice but could be evaluated for selected patients.*

### What Is the Role and Mode of Intraoperative Radiotherapy?

Intraoperative radiotherapy (IORT) was called as a valuable option for previously irradiated patients. Haddock et al^299^ reported their results of 10-20 Gy IORT in pelvic recurrences of rectal cancer with or without external beam radiation therapy (EBRT). Local control at 3 years was 0% with IORT alone and 30% with EBRT + IORT and survival improved from 12% to 38% with the addition of EBRT.^299^

The largest retrospective data on IORT was published by Mayo Clinic which included 607 locally advanced recurrent CRC patients.^300^ EBRT was mostly delivered preoperatively (median 45 Gy) with 5-FU, and the median IORT dose was 15 Gy. As 5-year OS was 34% in their series, IORT doses of 12.5 Gy or less were related to a 3% incidence of grade 2 or grade 3 neuropathy while doses of 15 Gy or higher were prominently associated with a 23% incidence of grade 2-3 neuropathy.^301^

A French multicenter phase-III trial from 1993 to 2001 randomized patients treated with preoperative EBRT to IORT or observation at the time of resection.^299,302^ Eligible candidates were patients with T3 (90%) or T4 primary rectal cancer or node-positive (34%) rectal cancer, treated with a preoperative external beam radiation dose of 40 Gy in 20 fractions and the IORT dose of 18 Gy. Local control at 5 years was 93% without IORT and 92% with IORT, while there was no significant difference in distant relapse, DFS and OS, or toxicity between the treatment groups.^299,302^ *Selection of patients is a key factor for appropriate use of IORT in the primary setting. Patients who might sound to benefit are those with T4 primaries and recurrent cancer with close margins or patients following preoperative chemoradiation with margins at risk for harboring undetectable residual disease. Besides*, *increased risk of neuropathy with IORT doses of 15 Gy or higher needs to be strongly considered in eligible patients for delivery.*

### Palliative Radiotherapy Indications

Locally advanced and recurrent rectal cancers frequently cause pelvic morbidity including pain, bleeding, and mass effect.^303^ Palliative pelvic RT is used to relieve these symptoms and delay local progression. There is no established optimal RT regimen, and clinical practices vary at treating institutions’ disposal. Overall, the symptom response rate to palliative RT in the retrospective series was 75%, and reported palliation rates were 78% for pain, 81% for bleeding and discharge, 71% for mass effect, and 72% for other pelvic symptoms. *Therefore*, *palliative pelvic RT for symptomatic rectal cancer appears to provide relief for a variety of pelvic symptoms*,* although there is no documented optimal RT regimen in this context.^303^*

#### Is There a Role of Stereotactic Body Radiotherapy or Stereotactic Ablative Radiotherapy for Metastases?

Takeda et al^304^ reported Japanese experience on the treatment outcomes of 21 patients (12 liver and 9 lungs) with 28 oligometastases from CRC treated by stereotactic ablative radiotherapy (SABR) using a risk-adapted regimen, from August 2011 to January 2015; a total dose of 50-60 Gy in 5 fractions was prescribed to the planning target volume. Along with the median follow-up duration of 27.5 months (range: 6.5-43.3 months), the local control rates at 1 and 2 years from the start of SABR were 100% without any severe toxicities (≥grade 3), while the DFS and actuarial OS rates were 62% and 55%, and 79% and 79%, respectively.^304^

Will Jin et al^305^ published a retrospective analysis of Georgetown University Hospital patients with oligoprogressive, metastatic CRC treated with stereotactic body radiation therapy (SBRT) between 2012 and 2016 and revealed 1-year local control of 82.9%, noting a distant first-site progression rate of 63.4%. Scorsetti et al^306^ published the preliminary results of their phase-II trial evaluating the feasibility of SBRT in the treatment of 61 patients having unresectable 1-3 liver metastases (45.9%, colorectal) with maximum individual tumor diameters less than 6 cm, between February 2010 and September 2011. After a median of 12 months (range: 2-26 months), the in-field local response rate was 94%, with no grade 3 or higher acute toxicity.

A recent phase II, open-label study called SABR-COMET^307^ enrolled 99 stage-4 patients from 4 different countries; of which, almost 20% with CRC were treated with a life expectancy of more than 6 months.^308^ The randomization was to either palliative RT or SABR. With a median follow-up time of 27 months, median OS was 41 months (95% CI = 26 months, upper limit not reached) for patients treated with SABR in comparison to 28 months (95% CI = 19-33 months) for standard palliative arm (stratified log-rank *P* = .09), as well as progression-free survival of 12 months (95% CI = 6.9-30 months) in SABR arm in comparison to 6 months (95% CI = 3.4-7.1 months) in standard palliative RT arm (*P* = .001).^308^ This is the first study to date revealing OS benefit by SABR in metastatic cancer patients, in addition to progression-free survival improvement. *Overall*,* SBRT/SABR seems to become an encouraging*,* non-invasive modality as an alternative to the surgical resection of oligometastases from CRCs, especially for patients who are not eligible for surgery.*

### Radiofrequency and Microwave Ablation

Patients with a limited number of lesions and involved sites should be considered as having oligometastatic disease. *The primary goal for patients who present with technically resectable liver metastases is R0 resection.* In these patients, the additional use of local ablation therapies such as radiofrequency ablation (RFA) has been shown to be feasible. The selection of the best technique from the list of ablative therapies for use in this setting differs according to the size and localization of the metastases, the rates of local control achieved (with the local control greater for surgery than for the other options), the invasiveness of the technique, the non-tumor-related prognostic considerations and patient factors, as well as patient preferences, the local expertise regarding the ablative methods, and consideration of patient frailty and life expectancy.^309^

A treatment goal for patients with metastatic CRC involves an attempt to eradicate all visible metastatic lesions using the best instrument from the toolbox of local ablative therapies, in combination with systemic therapy.^309^ The overall goal of this strategy is not necessarily to cure the patient, as the prognosis for these patients is generally poor due to the unfavorable localization of their metastases and the number of involved organs coupled with the limitations of local ablative treatments, compared with surgical resection. The CLOCC trial, a randomized phase-II trial with a median follow-up of 9.7 years, has demonstrated that aggressive local treatment can prolong OS in patients with unresectable colorectal liver metastases.^310^ In patients with advanced metastatic CRC, thermal ablation such as RFA often cannot be used due to the inherent size limitation of ∼3 cm. However, in the phase-II CLOCC trial (chemotherapy plus or minus RFA), RFA combined with surgical resection for the treatment of patients with CRC liver metastases suggested an improvement in both PFS and OS. A considerable amount of data are available on the use of thermal ablation in combination with liver resection for the treatment of patients with CRC liver metastases.

*In patients with only unresectable liver metastases*,* or oligometastatic disease*,* thermal ablation techniques such as RFA or microwave ablation can be considered. The decision should be taken by an MDTB based on institutional experience, tumor characteristics, and patient preference.*

### Radioembolization or Chemoembolization

To date, the data on chemoembolization for liver metastases from CRC are mostly observational series in various treatment situations.^311-313^ Comparative data are limited to irinotecan-based drug-eluting beads in a small phase-II cohort in previously treated patients showing a benefit vs systemic chemotherapy,^314^ and the role of intra-arterial irinotecan in patients pre-exposed to IV irinotecan is unclear.^309^

Radioembolization involves a single delivery of yttrium-90 connected to either resin or glass particles into the hepatic artery with the therapeutic effect essentially limited to irradiation. For patients with liver-limited metastases failing the available chemotherapeutic options, radioembolization with yttrium-90 resin microspheres has been shown to prolong the time to tumor progression in the liver, based on a small randomized phase-III study.^315^

*For patients with liver-limited disease failing the available chemotherapeutic options*,* radioembolization with yttrium-90 microspheres should be considered. If radioembolization is not possible for any reason*, *chemoembolization may be also considered a treatment option.*

#### Can Radioembolization Be Used as a Salvage Therapy in Patients with Liver Metastasis?

The use of radioembolization with resin microspheres has demonstrated improved results in the third-line or chemorefractory disease in patients with liver-dominant metastatic disease.^3^ The SIRFLOX and FOXFIRE studies failed to show improved OS with the combinational use of radioembolization (resin microspheres) with systemic chemotherapy compared to chemotherapy alone in the first-line treatment of patients with metastatic CRC with unresectable liver metastases.^316^

*Combination of Radioembolization with Chemotherapy in the First-Line Can Only Be Recommended in Clinical Trials. Radioembolization Should Be Considered in the Setting of Salvage Strategy After Being Discussed in MDTB.*

### Can Radioembolization Be Used for Downstaging of Liver Metastases?

The evidence for downstaging of metastatic liver disease with radioembolization is limited to a few case reports and small case series and 1 small clinical trial.^317^ Justinger et al^318^ reported 13 CRC patients with marginally resectable liver metastasis who were treated with resin microspheres for intended downstaging.^318^ Hepatic resection was performed in 11/13 patients after a median of 57 days (range: 39-153) following radioembolization, combined with associating liver partition and portal vein ligation for staged hepatectomy in 7/11 and with portal vein embolization in 1/11.

*Radioembolization for downstaging of liver metastases should be considered in the setting of salvage strategy after being discussed in MDTB.*

### Colorectal Cancer Screening and Surveillance

Characteristics of a successful CRC screening program should include effective identification of individuals eligible for screening, determination of a consistent screening strategy in the national setting, initiation of screening at the appropriate age, applicability and accessibility of screening tools, and follow-up of performance measures to ensure high-quality screening in the population. Colorectal cancer screening parameters include identification of eligible individuals, consistency of screening strategy at the national level, applicability/accessibility of screening tools, and performance measures. The performance of the screening program should be followed up and reported by the local and national regulatory authorities to ensure a high-quality screening process at the level of physicians and screening centers.^319^

Evidence-based quality indicators for a colonoscopy screening are as follows: The adenoma detection rate should be ≥25% overall or ≥30% for male patients and ≥20% for female patients. Cecal intubation rate should be ≥95%.^319,320^ Split-dosing of bowel preparations should be used to ensure effective cleansing of the colon before the colonoscopy procedure. The split-dose regimen is recommended because effective bowel preparation requires at least half the preparation to be ingested on the day of the colonoscopy.^321^

The National CRC Screening Program was initiated formally by the Ministry of Health in Turkey on September 1, 2014. *According to the national screening program, it is recommended to offer a fecal occult blood test (FOBT) every 2 years for every individual starting at the age of 50. Regardless of the initial FOBT result, the program mandates colonoscopy at the age of 51 and a follow-up colonoscopy 10 years after the initial negative colonoscopy.* Colorectal cancer screening protocols are highly variable around the world. While programmed screening is common among European countries, opportunistic screening using colonoscopy is the main strategy in the United States. In many European countries, including United Kingdom, screening is provided by primary care physicians using fecal immunochemical test (FIT) or FOBT. In several countries including Germany and Italy, colonoscopy is the preferred initial screening tool. The United States Preventive Services Task Force (USPSTF) guideline^322^ does not give preference for any single screening test over one another and advises that patients should be offered a choice among screening modalities including stool-based tests or direct colon visualization techniques. The stool-based test includes annual FOBT, annual FIT, or FIT-stool DNA every 1-3 years, while direct visualization techniques include flexible sigmoidoscopy alone (every 5 years), or combined (every 10 years), with the annual FIT; colonoscopy (every 10 years); and CT colonography (every 5 years). The Multi-Society Task Force of Colorectal Cancer, composed of the American College of Gastroenterology, the American Gastroenterological Association, and the American Society for Gastrointestinal Endoscopy, issued updated CRC screening guidelines in 2017.^319^ This multi-society guideline categorizes screening tests into 3 tiers. The most highly recommended is the first-tier which includes colonoscopy every 10 years or annual FIT. Second-tier includes CTC every 5 years, FIT-stool DNA every 3 years, and flexible sigmoidoscopy every 5-10 years. Third-tier is capsule colonoscopy every 5 years. The optimal screening method for CRC is colonoscopy; however, considering the limited financial resources and shortage in the number of endoscopists, annual FIT/FOBT should be incorporated as the main screening tool in the primary care setting. Nevertheless, primary care physicians should refer all individuals for colonoscopy starting at the age of 50 regardless of the FIT/FOBT result.

The international guidelines recommend a repeat colonoscopy after 3 years if index colonoscopy reveals a high-risk polyp (>3 adenomas or sessile serrated polyps, villous component, high-grade dysplasia or intramucosal cancer, any polyp ≥10 mm). Individuals with high-risk features (personal or family history of CRC, inflammatory bowel disease, and hereditary cancer syndromes) require earlier initiation of screening with shorter interval; however, details of screening algorithm in high-risk individuals are beyond the scope of this consensus report. The National CRC Screening Program algorithm is appropriate and recommendations from the USPSTF 2012 guideline, which can be summarized as 3-5-10 years intervals for colonoscopy according to the result of index colonoscopy, should be implemented into the current screening program.

### What Is the Optimal Age Interval for CRC Screening?

Most of the societies, except the American Cancer Society (ACS), recommend initiating CRC screening at the age of 50 in all individuals with average risk. The ACS updated its guidelines for screening people at average risk for CRC at age 45 years and above. The rationale for a younger starting age for screening is the apparent increased incidence of CRC in younger adults.^323^ Both USPSTF and ACS guidelines recommend continuing screening up to age 75 years if screening is up-to-date. If screening is not up-to-date, they consider screening up to age 85 years. It is recommended to discontinue screening in patients over 85 years of age and patients with a shortened life expectancy which is defined as less than 10 years of remaining life.

*We recommend initiating CRC screening at the age of 50 in individuals with average risk and should be continued till 75 years. Colorectal cancer screening should be initiated at the age of 40 or earlier in patients with high-risk features. The timing should be individualized according to the type of risk. The decision of continuing CRC screening at the age of 76-85 years should be individualized according to the person’s clinical characteristics and expectations. Colorectal cancer screening over 85 years of age should be discouraged.*

#### Which of the Imaging Technic and Screening Method Is Cost-Effective?

*Brachytherapy* and CEA follow-ups were found to be more cost-effective.^324^ Contrast-enhanced MRI seems to be more cost-effective for detecting metastases that are undetectable with other imaging techniques for deciding treatment to curative intent for patients who are scheduled to undergo liver resection. Magnetic resonance imaging has also been found more precise in early and correct diagnosis.^325^

*We recommend performing CEA every 3-6 months in first 2 years and then every 6 months for a total of 5 years.*

*We recommend performing thoraco-abdominal CT as per the following duration:*

*For stage II and III disease, every 6-12 months for a total of 5 years,*

*For stage IV disease, every 3-6 months for 2 years then every 6-12 months for a total of 5 years.*

*We recommend performing colonoscopy in 1 year except if no preoperative colonoscopy was done due to obstructing lesion; in 3-6 months if advanced adenoma is present and to repeat it in 1 year; and if no advanced adenoma is present, repeat it in 3 years and then every 5 year.*

*Fluorodeoxyglucose positron emission tomography/computed tomography is not indicated for routine follow-up and can be performed selectively.*

#### Anorectal functions, Life Quality, and Fertility During Rectal Cancer Treatment

Better functional outcomes provide better life quality in rectal cancer patients under treatment and follow-up. Functional advantages that have often been associated with preoperative RT, as opposed to RT given postoperatively, are related to both tumor response and preservation of normal tissue.^326^ Thus, advantages of the neoadjuvant approach include better local control (even in the setting of optimal TME, an increased likelihood of sphincter-saving surgery, a lower risk of posttreatment bowel dysfunction (soiling and frequent stooling) and a lower risk of chronic anastomotic stricture. Also, preoperative RT can avoid the occurrence of RT-induced injury to the small bowel trapped in the pelvis by postsurgical adhesions.^134^

All therapeutic options for rectal cancer, namely surgery, chemotherapy, and radiation therapy, adversely affect fertility. Discussions about fertility risks associated with CRC treatment occur infrequently (34%) among young adults with newly diagnosed CRC.^327^ However, it has been shown that great majority of younger cancer survivors see their cancer experience as potentially making them better parents. Those without children may want to have children in the future.^328^ A study among younger male cancer survivors showed positive emotional effects in cancer therapies when they are offered banking sperm even if it will never be used.^329^ *Thus, in rectal cancer fertile patients in whom surgery, chemotherapy, and RT are planned, fertility risks should be counseled and options to protect fertility should be offered. These options include oocytes and embryo cryopreservation, ovarian transposition and ovarian suppression in females, and cryopreservation “bank” sperm in males.*

Although there is no data on preoperative assessment of preoperative sphincteric functions in rectal cancer patients who do not demonstrate signs of incontinence and/or pudendal neuropathy, a study has shown that RT can cause significant prolongation of pudendal nerve terminal motor latency (PNTML).^330^ *So, if a patient develops symptoms or signs of incontinence after preoperative CRT while waiting for rectal surgery, a PNTML test can be conducted to determine the surgical technique for the cure and provide good life quality.*

*No pretreatment continence assessment will be necessary in rectal cancer patients who do not have incontinence signs and symptoms. However, sphincteric functions should be determined by manometry, EUS, and PNTML in patients with any signs of incontinence.^331^*

### Cancer Risks, Associated Genes, and Recommendations for Management of Hereditary Colorectal Cancer

Hereditary CRCs occurring due to mutations and defects in certain genes make up roughly 5% of all CRC. In addition, about 25%-30% of CRC patients might have a family member with a diagnosis of CRC without any known genetic alterations. High-risk hereditary predisposition syndromes have been associated with a 70%-100% lifetime risk for development of CRCs and many syndromes carry an increased risk for extra-intestinal malignancies. Detection of these patients by family history and appropriate genetic tests give individual cancer risk determination, appropriate cancer screening, follow-up, and prevention options. The role of genetic counseling is recommended in managing these high-risk persons.

### Hereditary CRC has 2 well-described forms:

1. Polyposis (including familial adenomatous polyposis (FAP) and attenuated FAP (AFAP), which are caused by pathogenic variants in the *APC* gene, and MUTYH-associated polyposis, which is caused by pathogenic variants in the *MUTYH* gene, and

2. LS (often referred to as hereditary nonpolyposis colorectal cancer), which is caused by germline pathogenic variants in DNA mismatch repair genes (*MLH1*, *MSH2*, *MSH6*, and *PMS2*) and *EPCAM*.

Many of these syndromes are also associated with extracolonic cancers and other manifestations. Serrated polyposis syndrome, which is characterized by the appearance of hyperplastic polyps, appears to have a familial component, but the genetic basis remains unknown. The natural history of some of these syndromes is still being described. Many other families exhibit aggregation of CRC and/or adenomas but with no apparent association with an identifiable hereditary syndrome and are known collectively as familial CRC. In addition, most individuals with CRC diagnosed before 50 years of age and without a family history of cancer do not have a pathogenic variant associated with an inherited cancer syndrome.

*Table 2* *combines genetic risks and our recommendations for testing strategies and genetic counseling with clinical screening and treatment measures under the guidance of the international guidelines.*

#### Definition of Hereditary Predisposition to Rectal Cancer and Genetic Counseling

Genetic testing for germline pathogenic variants in *MLH1*, *MSH2*, *MSH6*, *PMS2*, and *EPCAM* help articulate appropriate intervention strategies for the LS-affected variant-positive individual and at-risk family members; APC gene testing needs to be warranted for FAP-predisposed families (Table 3).

If a pathogenic variant is identified in an affected person, then testing for that same pathogenic variant should be offered to all at-risk family members. At-risk relatives who test negative for the identified pathogenic variant in the family are not at increased risk of CRC or other LS-associated malignancies and can follow surveillance recommendations applicable to the general population. Family members who carry the familial pathogenic variant need to be referred to surveillance and management.

Colonoscopy for CRC screening and surveillance is commonly performed in individuals with hereditary CRC syndromes and has been associated with improved survival outcomes. *Surveillance of LS patients with colonoscopy every 1-2 years has been shown to reduce CRC incidence and mortality.*

*Prophylactic surgery (colectomy) has also been shown to improve survival in patients with FAP. The timing and extent of risk-reducing surgery usually depend on the number of polyps, size, histology, and symptomatology.* For patients with LS and a diagnosis of CRC, extended resection is associated with fewer metachronous CRCs and additional surgical procedures for colorectal neoplasia than in patients who undergo segmental resection for CRC. *The surgical decision must take into account the age of the patient, comorbidities, clinical stage of the tumor, sphincter function, and individual consultation.*

## Figures and Tables

**Table 1.** Criteria for Low- or High-Risk Rectal Cancer and Lymph Node Metastasis

Low risk	High risk
Well differentiated	Poorly differentiated
Size <3cm	Size ≥3 cm
Circumferential involvement <30% of lumen	Circumferential involvement ≥30% of lumen
Superficial involvement (SM1)	Deep layer involvement (SM2-SM3)
Margins ≥2 mm	Margins <2 mm
No lymphovascular invasion No tumor budding No perineural invasion No lymphocytic invasion	Lymphovascular invasion Tumor budding Perineural invasion Lymphocytic invasion

SM, submucosal invasion; SM1, invasion into the upper third of the submucosa; SM2, invasion into the middle third of the submucosa; SM3, invasion into the lower third of the submucosa.

**Table 2.** Cancer Risks, Genes Associated, and Recommendations for Management of Hereditary CRC Syndromes

Syndrome	Gene (s)	Lifetime Cancer Risks	(95% CI)	Screening/Surveillance	Preventative Surgery
**Lynch syndrome**	MSH2	Colorectum	49 (29-85)	Colonoscopy every 1-2 years starting at age 20-25 years	Consider prophylactic hysterectomy once childbearing completes
**Lynch syndrome**	EPCAM	Endometrium Stomach Ovary Hepatobiliary Upper urinary tract Pancreas Small bowel CNS (glioblastoma)	57 (22-82) 11-19 20 (1-66) 2-7 4-5 3-4 1-4 1-3	Consider upper endoscopy every 3-5 years starting at age 30-35 years Consider endometrial cancer screening	
**Lynch syndrome**	MLH1	Colorectum Endometrium Stomach Ovary Hepatobiliary Upper urinary tract Pancreas Small bowel CNS (glioblastoma)	52 (31-90) 21 (9-82) 11-19 38 (3-81) 2-7 4-5 3-4 1-4 1-3	Colonoscopy every 1-2 years starting at age 20-25 years Consider upper endoscopy every 3-5 years starting at age 30-35 years Consider endometrial cancer screening	Consider prophylactic hysterectomy once childbearing completes
**Lynch syndrome**	MSH6	Colorectum Endometrium Stomach Ovary Urinary tract	18 (13-30) 17 (8-47) ≤31 (0-3) <1	Colonoscopy every 1-2 years starting at age 20-25 years Consider upper endoscopy every 3-5 years starting at age 30-35 years Consider endometrial cancer screening	Consider prophylactic hysterectomy once childbearing completes
**Lynch syndrome**	PMS2	Colorectum Endometrium	15-20 15	Colonoscopy every 1-2 years starting at age 20-25 years Consider upper endoscopy every 3-5 years starting at age 30-35 years Consider endometrial cancer screening	Consider prophylactic hysterectomy once childbearing completes
**FAP: Classic**	APC	Colorectum Duodenum/ periampullary	100 4-12	Colonoscopy every 1-2 years starting at age 10-12 years Upper endoscopy every 1-3 years starting at age 18-25 years	Consider colectomy when polyp burden is too great for endoscopic control

**Table 3.** Criteria that Warrant Assessment for CRC Syndromes Predisposition

Cancer/Feature	When to Refer to Genetic Counseling	Syndrome(s) to Consider
Colorectal cancer	Colorectal cancer dx at age <50	LS, OMIM 120435, 120436; CMMRD, OMIM 276300; MAP, OMIM 608456
	Colorectal cancer dx at age ≥50 if there is a first-degree relative with colorectal or endometrial cancer at any age	
	Synchronous or metachronous colorectal or endometrial cancers in the same person	
	Colorectal cancer showing mismatch repair deficiency on tumor screening in the same person or in close relatives	
	Colorectal cancer and 2 additional Cowden syndrome criteria in the same person	Cowden, OMIM 158350
	Colorectal cancer and 1 additional LFS tumor in the same person or in 2 relatives, 1 dx at age ≤45	LFS, OMIM 151623
	Colorectal cancer with ≥10 cumulative adenomatous colon polyps in the same person	FAP, OMIM 175100; MAP, OMIM 608456
Colorectal polyposis, adenomatous	≥10 cumulative adenomatous colon polyps in the same person	FAP, OMIM 175100; MAP, OMIM 608456
Colorectal polyposis, hamartomatous	3-5 cumulative histologically proven juvenile polyps in the same person	JPS, OMIM 174900
	Multiple juvenile polyps throughout the GI tract in the same person	
	Any number of juvenile polyps with a positive family history of JPS	
	≥2 cumulative histologically proven PJ polyps in the same person	PJS, OMIM 175200
	≥1 PJ polyp and mucocutaneous hyperpigmentation in the same person	
	Any number of PJ polyps and a positive family history of PJS	
	GI hamartoma or ganglioneuroma and 2 additional Cowden syndrome criteria in the same person	Cowden, OMIM 158350
	Rectal hamartomatous polyps and 1 additional TSC criterion in the same person	TSC, OMIM 191100
	Diffuse ganglioneuromatosis of the GI tract	MEN2, OMIM 171400
Colorectal polyposis, serrated	≥5 SPs proximal to the sigmoid colon, 2 of which are >1 cm in diameter, in the same person	SPS, not in OMIM
	>20 SPs at any site in the large bowel in the same person	
	Any number of SPs proximal to the sigmoid colon and a positive family history of SPS	
Colorectal polyposis, mixed	≥10 cumulative polyps with >1 histology in the same person	HMPS, OMIM 201228, 610069

CMMRD, constitutional mismatch repair deficiency; FAP, familial adenomatous polyposis; HMPS, hereditary mixed polyposis syndrome; JPS, juvenile polyposis syndrome; LFS, Li-Fraumeni syndrome; LS, Lynch syndrome; MAP, MUTYH-associated adenomatous polyposis; MEN, multiple endocrine neoplasia; OMIM, Online Mendelian Inheritance in Man; PJS, Peutz–Jeghers syndrome; SPS, Stiff-person syndrome; TSC, tuberous sclerosis complex; GI, gastrointestinal tract.

## The members of the consensus faculty

### (The İstanbul Group)

Abdullah Zorluoğlu

Adil Baykan

Afag Aghayeva

Ahmet Çakmak

Ahmet Karaman

Ali Arıcan

Alper Doğruöz

Alper Sonkaya

Anıl Ergin

Arda Ulaş Mutlu

Arzu Tiftikçi

Aslıhan Güven Mert

Atakan Demir

Aydan Arslan

Ayşe Altınok

Ayşe Armutlu

Ayşegül Bağda

Bahadır Bozkırlı

Bahattin Çiçek

Banu Atalar

Bengisu Yılmaz

Bilgehan Şahin

Buket Bayram Kuvvet

Bülent Boran

Bülent Gürbüz

Burak Kılıçarslan

Çağrı Bilgiç

Cahide İnci Şahin

Can Çalışkan

Cengiz Yakıcıer

Cevriya Nida Dönmez

Çiğdem Usul Afşar

Davut Şahin

Deniz Atasoy

Duygu Sezen

Ebru Kırbıyık

Emel Ahıshalı

Emre Altınmakas

Enis Özyar

Ercan Karaarslan

Erdoğan Süleyman

Eren Kaya

Erman Sabutay

Eşref Kızılkaya

Eylem Akar

Farid Babashov

Fatih Selçuk Biricik

Fuat Barış Bengür

Fulya Ağaoğlu

Hakan Koyuncu

Hakan Ümit Ünal

Hülya Kozalı

İbrahim Yıldız

Işık Aslay

İsmail Ahmet Bilgin

Kardelen Uzun

Kerim Kaban

Koray Güven

Mehmet Mahir Fersahoğlu

Mehmet Teomete

Meriç Şengöz

Metin Kanıtez

Mustafa Bozkurt

Mustafa Uygar Kalaylcı

Nilsu Güvençer

Nilüfer Kılıç Turankuş

Nurten Türel Küçükmetin

Onur Bayram

Orhan Ağcaoğlu

Orhun Çıg Taşkın

Özdal Ersoy

Özde Melisa Celayir

Özlem Sönmez

Şahika Mert

Sami Açar

Serap Yücel

Seray Uğur

Serhat Meriç

Serkan Zenger

Serpil Yılmaz

Sezer Sağlam

Sibel Yayla

Şule Karaman

Süleyman Alıcı

Süleyman Orman

Suna Yapalı

Tamer Aksoy

Taner Korkmaz

Tarık Akçal

Türker Ertürk

Ümmihan Topal

Volkan Adsay

Volkan Tumay

Yasin Kara

Zeliha Füsün Baba

Zeynep Güral
